# Addressing the clinical unmet needs in primary Sjögren’s Syndrome through the sharing, harmonization and federated analysis of 21 European cohorts

**DOI:** 10.1016/j.csbj.2022.01.002

**Published:** 2022-01-07

**Authors:** Vasileios C. Pezoulas, Andreas Goules, Fanis Kalatzis, Luke Chatzis, Konstantina D. Kourou, Aliki Venetsanopoulou, Themis P. Exarchos, Saviana Gandolfo, Konstantinos Votis, Evi Zampeli, Jan Burmeister, Thorsten May, Manuel Marcelino Pérez, Iryna Lishchuk, Thymios Chondrogiannis, Vassiliki Andronikou, Theodora Varvarigou, Nenad Filipovic, Manolis Tsiknakis, Chiara Baldini, Michele Bombardieri, Hendrika Bootsma, Simon J. Bowman, Muhammad Shahnawaz Soyfoo, Dorian Parisis, Christine Delporte, Valérie Devauchelle-Pensec, Jacques-Olivier Pers, Thomas Dörner, Elena Bartoloni, Roberto Gerli, Roberto Giacomelli, Roland Jonsson, Wan-Fai Ng, Roberta Priori, Manuel Ramos-Casals, Kathy Sivils, Fotini Skopouli, Witte Torsten, Joel A. G. van Roon, Mariette Xavier, Salvatore De Vita, Athanasios G. Tzioufas, Dimitrios I. Fotiadis

**Affiliations:** a Unit of Medical Technology and Intelligent Information Systems, Dept. of Materials Science and Engineering, University of Ioannina, Ioannina, Greece; b Dept. of Pathophysiology, School of Medicine, University of Athens, Athens, Greece; c University Hospital of Ioannina, Ioannina, Greece; d Dept. of Informatics, Ionian University, Corfu, Greece; e Clinic of Rheumatology, Dept. of Medical and Biological Sciences, Udine University, Udine, Italy; f Centre for Research and Technology Hellas, Thermi, Thessaloniki, Greece; g Institute for Systemic Autoimmune and Neurological Diseases, Athens, Greece; h Fraunhofer Institute for Computer Graphics Research IGD, Darmstadt, Germany; i ATOS, Madrid, Spain; j Institute of Legal Informatics, Leibniz Universität Hannover, Hannover, Germany; k Institute of Communication and Computer Systems, School of Electrical and Computer Engineering, National and Technical University of Athens, Athens, Greece; l Bioengineering Research and Development Center, Faculty of Engineering, University of Kragujevac, Kragujevac, Serbia; m Biomedical Informatics and eHealth Laboratory, Dept. of Electrical and Computer Engineering, Hellenic Mediterranean University, Heraklion, Greece; n Dept. of Clinical and Experimental Medicine, University of Pisa, Pisa, Italy; o Centre for Experimental Medicine and Rheumatology, William Harvey Research Institute, Queen Mary University of London and Barts’ Health NHS Trust, London, United Kingdom; p Dept. of Rheumatology and Clinical Immunology, University of Groningen, University Medical Center Groningen, the Netherlands; q Rheumatology Dept., University Hospitals Birmingham NHS Foundation Trust, Birmingham, UK; r Dept. of Rheumatology, Erasme Hospital, Université Libre de Bruxelles, Brussels, Belgium; s Laboratory of Pathophysiological Biochemistry and Nutrition, Université Libre de Bruxelles, Brussels, Belgium; t Univ Brest, Inserm, CHU de Brest, UMR1227, Lymphocytes B et Autoimmunité, Brest, France; u Dept. of Rheumatology and Clinical Immunology, Charité-Universitätsmedizin Berlin, Berlin, Germany; v Rheumatology Unit, Dept. of Medicine and Surgery, University of Perugia, Perugia, Italy; w Division of Rheumatology, Dept. of Biotechnological and Applied Clinical Sciences, University of L'Aquila, L'Aquila, Italy; x Dept. of Clinical Science, University of Bergen, Bergen, Norway; y Institute of Cellular Medicine, Newcastle University, Newcastle upon Tyne, UK; z Dept. of Internal Medicine and Medical Specialties, Rheumatology Clinic, Sapienza University of Rome, Rome, Italy; aa Laboratory of Autoimmune Diseases Josep Font, IDIBAPS-CELLEX, Barcelona, Spain; ab Oklahoma Medical Research Foundation, OK, US; ac Dept. of Internal Medicine and Clinical Immunology, Euroclinic Hospital, Athens, Greece; ad Dept. of Rheumatology and Immunology, Hanover Medical School, Hanover, Germany; ae Dept. of Rheumatology and Clinical Immunology, University Medical Center Utrecht, Utrecht University, Utrecht, the Netherlands; af Dept. of Rheumatology, Hôpital Bicêtre, Assistance Publique-Hôpitaux de Paris, Paris, France; ag Dept. of Biomedical Research, FORTH-IMBB, Ioannina, Greece

**Keywords:** Data sharing, Data curation, Data harmonization, Federated AI, Lymphoma classification, Biomarkers, Primary Sjögren’s syndrome

## Abstract

For many decades, the clinical unmet needs of primary Sjögren’s Syndrome (pSS) have been left unresolved due to the rareness of the disease and the complexity of the underlying pathogenic mechanisms, including the pSS-associated lymphomagenesis process. Here, we present the HarmonicSS cloud-computing exemplar which offers beyond the state-of-the-art data analytics services to address the pSS clinical unmet needs, including the development of lymphoma classification models and the identification of biomarkers for lymphomagenesis. The users of the platform have been able to successfully interlink, curate, and harmonize 21 regional, national, and international European cohorts of 7,551 pSS patients with respect to the ethical and legal issues for data sharing. Federated AI algorithms were trained across the harmonized databases, with reduced execution time complexity, yielding robust lymphoma classification models with 85% accuracy, 81.25% sensitivity, 85.4% specificity along with 5 biomarkers for lymphoma development. To our knowledge, this is the first GDPR compliant platform that provides federated AI services to address the pSS clinical unmet needs.

## Highlights

• Data sharing can address open issues and clinical unmet needs in rare diseases.
• Data curation enhanced the cohort data quality in primary Sjögrens Syndrome (pSS).
• Semantic analysis yielded 7,156 harmonized patients across 21 cohorts in pSS.
• Federated tree ensembles yield explainable AI models for lymphoma development.
• Salivary gland swelling & cryoglobulinemia increase the risk for lymphomagenesis.

## Graphical abstract

**Figure:**
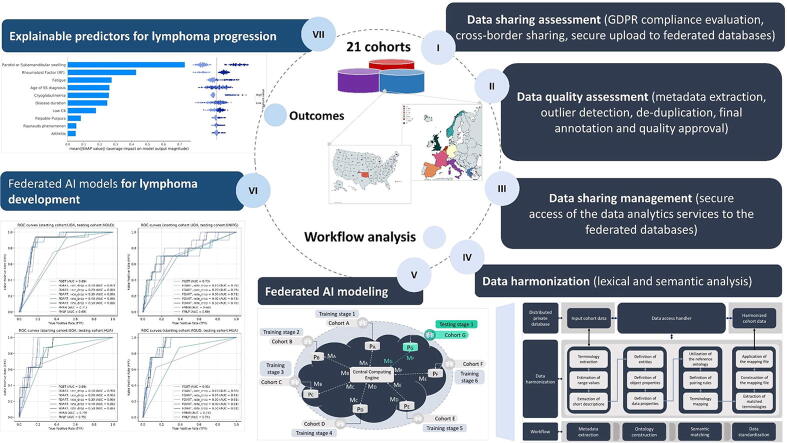

## 1 Introduction

Primary Sjögren’s Syndrome (pSS) is a chronic systemic autoimmune disease which is characterized by a wide spectrum of clinical manifestations varying from mild disease limited to exocrine glands to severe multi-systemic involvement [1], [2], [3]. According to the literature [1], [2], [3], [4], [5], pSS has the most unbalanced gender ratio with almost 10 females affected per 1 male while the development of B-cell non-Hodgkin lymphoma (NHL) complicates about 5% of patients during the disease course [1], [2], [3], [4], [5]. Female preponderance, *peri*-epithelial lymphocytic infiltration of the affected organs, B-cell hyperactivity manifested as hypergammaglobulinemia, as well as, activation of interferon and B-cell activating factor pathways are considered hallmarks of the disease. Although the cause of pSS remains unknown, the disease develops in the context of genetic, environmental, and immune factors. In fact, pSS is unique not only due to its clinical impact but also as one of the few disease “models” linking autoimmunity with cancer and especially lymphoproliferative disorders. As in other systemic autoimmune or neoplastic diseases, the lack of patient stratification models in pSS: (i) increases the risk of producing unsatisfactory or sub-optimal results in clinical trials employing novel and expensive drugs, and (ii) hampers the definition of evidence-based health policies. These two issues are related with the unmet needs in pSS which involve the development of robust lymphoma classification models and the extraction of biomarkers.

Only a few relevant studies have been reported in the literature concerning the design and application of lymphoma classification models, as well as, the discovery of biomarkers for lymphoma development and progression. Most of these studies adopt univariate and multivariate statistical methods [6], [7], [8] to identify independent factors for lymphoma development which in turn are utilized as independent variables for regression analysis with the dependent variable being set to lymphoma. A more straightforward method for the detection of risk factors was presented in two studies [9], [10], where the fast correlation-based filter selection (FCBF) method was deployed to identify robust independent factors for lymphoma development, following a logistic regression analysis. Furthermore, supervised machine learning methods [11], [12], [13], [14], [15], such as, the supervised tree ensembles, the Support Vector Machines (SVMs), and the artificial neural networks (ANNs) have been utilized in the literature for the development of robust lymphoma classification models in pSS with adequate performance. However, these studies have poor statistical power due to the reduced population size, since they adopt either a single cohort analysis approach in [11], [12], [13] or a small-scale but straightforward analysis typically involving no more than four cohorts in [14], [15].

The reduced quality and the structural heterogeneity of the existing cohort databases along with the lack of data curation pipelines obscure the development of robust AI models and the detection of biomarkers. According to the literature, the existing platforms and tools for data curation focus on the development of qualitative approaches, such as, the ExeTera software [16] which provides data filtering options based on semantic information, and the dementias platform UK (DPUK), where emphasis is given on the definition of standard data quality criteria [17]. Regarding data harmonization, the existing tools are semi-automated and disease-specific, focusing on the extensive collaboration of the clinician with the technical experts. The DataSHaPER [18] uses a DataSchema as a reference model to harmonize heterogeneous data structures through the manual definition of elements and rules for terminology mapping across biobanks. Furthermore, the BiobankConnect software [19] points particular emphasis on the application of lexical matching to identify lexically matched terminologies across biobanks. The SORTA tool [20] utilizes ontologies to align terminologies with conceptual similarity across diverse ontologies and particularly in biobanks.

Apart from data harmonization, the lack of GDPR compliant and cross border data sharing mechanisms has a direct effect on the statistical power of the cohort studies. The conventional data integration strategy, where patients’ data from different clinical centers are integrated into a centralized database is not always feasible neither viable due to legal violations and security compromise attempts that will expose the patient data. The euroCAT platform [21], [22] offers a distributed learning framework for the development of multi-centric models through the installation of local servers on the hospital’s premises. The PHT platform [23] adopts a similar methodology for distributed analysis through the training of distributed logistic regression models with adequate performance. In another study, four cohorts were analyzed in a distributed manner yielding lymphoma classification models with more than 85% sensitivity and specificity but with reduced statistical power [15]. Thus, the existence of a platform which adopts a federated data management strategy that avoids the installation of local servers in the hospitals’ premises and enables the sharing of sensitive data from multiple cohorts with heterogeneous structure remains a crucial challenge.

Towards this direction, we present the HarmonicSS platform, a highly scalable and GDPR compliant cloud computing infrastructure which offers beyond the state-of-the-art services for federated data storage, curation, and harmonization, as well as, trustworthy and explainable federated AI (Artificial Intelligence) modeling workflows, which are in line with the EU data protection regulations for novel infrastructures, data spaces, data platforms and AI tools [24], [25]. Τhe platform was developed under the HarmonicSS EU funded project (HARMONIzation and integrative analysis of regional, national and international Cohorts on primary Sjögren’s Syndrome (pSS) towards improved stratification, treatment and health policy making) [7], [9], [10], [11], [12], [14], [15] and removes the need for the installation of local servers or any type of software in each site through the adoption of a federated data management platform which supports a large family of federated AI algorithms yielding interpretable and explainable AI models. Data curation workflows are utilized on the cohort data to enhance their quality along with lexical and semantic interlinking mechanisms to enable data harmonization. A large-scale case study was conducted to address the clinical unmet needs in pSS through the federated analysis of 21 European cohorts on pSS. Through the platform, the users were able to curate and harmonize 7,156 patient records yielding robust, explainable, and trustworthy AI models for lymphoma classification along with five biomarkers for lymphoma development with small execution time complexity. To our knowledge, this is the first GDPR compliant and federated cloud computing platform which provides easy to use services, to address the clinical unmet needs in pSS.

## 2 Materials and methods

### 2.1 Overview

**Fig. 1:**
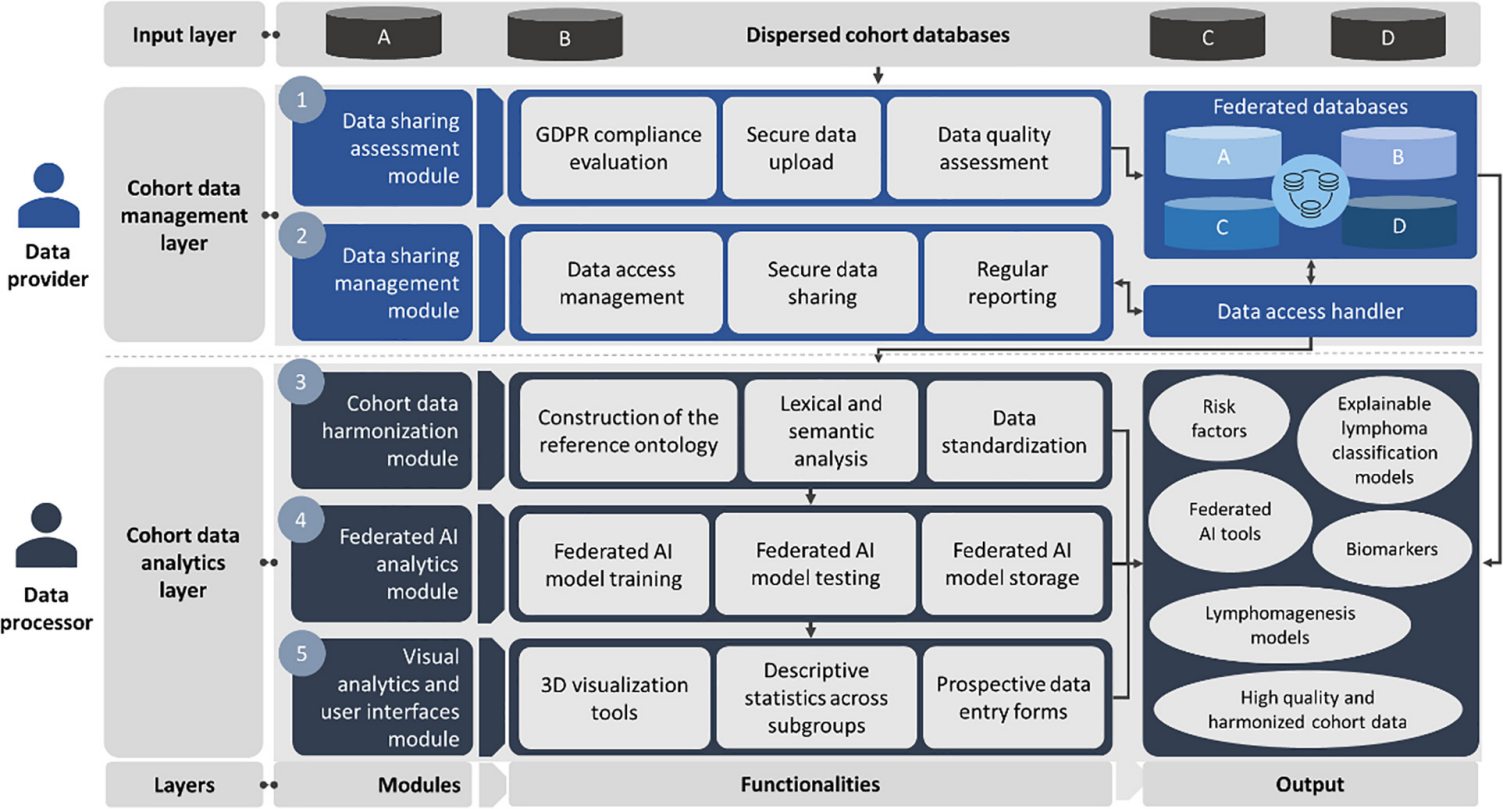
An illustration of the core modules of the HarmonicSS cloud computing platform.

The HarmonicSS platform includes a wealth of harmonized cohort databases on top of which the core modules operate. The main architectural components (or core modules) of the HarmonicSS platform have been designed according to a hierarchical, top-down approach (Fig. 1) and have been grouped into three layers, namely: (i) the input layer, (ii) the cohort data management layer, and (iii) the cohort data analytics layer. The main users of the HarmonicSS platform are the data provider and the data processor. The data provider interacts with the data management layer which is located on the top of the architecture and includes: (i) the data sharing assessment module, and (ii) the data sharing management module. On the other hand, the data processor interacts with the data analytics layer which is located at the bottom of the architecture and includes: (i) the cohort data harmonization module, (ii) the federated AI analytics module, and (iii) the visual analytics and user interfaces module. Depending on the type of functionality each layer offers, the modules can be also grouped into two main categories, namely the cohort data governance modules and the cohort data analytics modules. The former includes the data sharing assessment and data sharing management modules, whereas the cohort data analytics modules include the cohort data harmonization, federated AI analytics, and visual analytics and user interfaces modules.

The data sharing assessment module ensures the GDPR (General Data Protection Regulation) compliance of the cohort data through the evaluation of the legal and ethical documents of the data providers who are interested in sharing their data with the HarmonicSS private cloud repositories. Once the GDPR compliance is ensured, a data curation workflow is applied to enhance the quality of the cohort data in terms of accuracy, relevance, and completeness. The data sharing management module offers a “handshaking” process which controls the access of the data analytics services into the private cloud databases. The cohort data harmonization module includes a series of mechanisms for the alignment of the heterogeneous structures of the curated cohort data according to the pSS reference model using semantic matching methods for cohort data transformation and ontology alignment based on a knowledge base. The federated AI analytics module supports the training of supervised machine learning algorithms across the federated cloud databases towards the construction of explainable and trustworthy AI models which are validated on a series of federated and harmonized testing cohort databases.

The outcomes of the modules from the cohort data management layer and the cohort data analytics layer are presented to the users of the platform through the visual analytics and user interfaces module. The latter provide highly interactive graphical user interfaces (GUIs) and visual analytics services, including 3D visualization methods based on graph theory and query-based descriptive statistics for subgroup analysis, as well as, prospective data entry forms which have been properly designed to enable the inclusion of prospective patient data automatically interlinked with the corresponding harmonized retrospective data on the federated databases. The HarmonicSS federated database management system is compatible with HL-7 (Health Level 7) data exchange protocols, including the FHIR (Fast Healthcare Interoperability Resources) [26] which enhances its adaptability and enables the interlinking of the HarmonicSS data with existing FHIR database management systems. In addition, the harmonized cohort data meet the principles of findability, accessibility, interoperability, and usability (FAIR) [27] and thus can serve as an interconnection hub with EOSC (European Open Science Cloud) initiatives and European data spaces for hosting and processing to support research in autoimmune diseases. The data management system avoids the use of centralized repositories and data lakes to avoid the compromise of sensitive data during a privacy breach and ensure the legal and ethical compliance of data sharing according to the GDPR.

### 2.2 Data sharing assessment module

#### 2.2.1 GDPR compliance evaluation (and cross-border sharing)

The data governance framework of the HarmonicSS platform puts particular emphasis on the legal and ethical compliance of patient’s data to fulfill the data protection regulations posed by the General Data Protection Regulation (GDPR) [28]. Towards this direction, a Data Controllers Committee (DCC) consisting of three technical and clinical experts was designated to supervise the GDPR compliance of the legal and ethical documents. These documents consist of a data protection impact assessment (DPIA) and a data protection agreement (DPA). The GDPR evaluation process is in concordance with the Article 5 of the GDPR, according to which the personal data are: (i) processed with respect to the legal regulations and in a secure way, (ii) adequate, (iii) accurate, and (iv) kept in such a form that they can be identified. The GDPR compliance process is in line with the Articles 16–22 of the GDPR [29] according to which the data subjects of the HarmonicSS platform have: (i) the right to be informed about the actions and participate in any automated decision which involves their data, (ii) the right to be forgotten, (iii) the right to object and restrict the processing of their data, and (iv) the right for data portability. Apart from the GDPR compliance, the HarmonicSS platform has enabled the cross-border sharing of genetic data samples from the U.S. and particularly from the OMRF (Oklahoma Medical Research Foundation) through the data sharing assessment procedure which controls access to sensitive data according to the HIPAA rules (Health Insurance Portability and Accountability Act) [30]. The data controllers and the data processors prepare codes of conduct with respect to: (i) the collection of personal data, (ii) the pseudonymization of personal data (data protection by design), (iii) the legitimate interests pursued by the data controllers, (iv) the transparency and fairness in data processing, and (v) data minimization (data protection by default), among others. The platform poses rules on data de-identification giving emphasis on protection safeguards, such as, data minimization, consent forms, employment of data protection officers for ensuring the GDPR compliance, all of them with respect to the individual rights who has the right to be forgotten. All patients who participated in the HarmonicSS project fulfilled the 2016 EULAR/ACR classification criteria for Sjogren’s syndrome as described in Shiboski et al. [31].

#### 2.2.2 Secure sharing of the cohort data

Upon the GDPR compliance of the DPIA and DPA documents, the pseudonymized patient data are uploaded into secure private databases within the Greek Research and Technology Network (GRNET) cloud infrastructure. The NextCloud [32] file hosting service was used to develop the federated database management system which was built on top of the cloud infrastructure to provide private cloud databases (and private cloud spaces, as well) for each data provider, as well as, to ensure the secure access of the federated data analytics services to each private cloud database. Through the NextCloud [32], the services of the data sharing assessment module can be easily integrated into any cloud infrastructure that fulfills the legal and ethical criteria for data sharing. Both the raw cohort data and the curated cohort data, as well as, the harmonized cohort data are stored in these private cloud databases. The data providers manage their private cloud spaces, similarly to the Google Drive, for personal use. SSL/TLS communication protocols were used for the communication with the private database of each data provider.

#### 2.2.3 Data quality assessment

##### 2.2.3.1 Metadata extraction and outlier detection

Useful metadata were automatically extracted from the 21 cohort databases, regarding the names of the available features and the value ranges, followed by a short description of the clinical domain knowledge. Then, the data curation workflow ensures that the structure of each shared dataset fulfills the following requirements: (i) the shared pseudonymized data are stored in a tabular format, (ii) each row in the tabular format corresponds to a patient record, and (iii) each column in the tabular format corresponds to a feature (e.g., a laboratory examination). The outlier detection stage of the data curation workflow involves the accurate detection and subsequent elimination of feature values that significantly deviate from the standard distribution of the clinical data either on a univariate or on a multivariate level. The univariate methods involve the application of the z-score and the Interquartile Range (IQR) [33] measures. The multivariate outlier detection methods involve the application of the isolation forests [34], [35], [36] and the local outlier factor (LOF) [34], [35], [36]. Isolation trees are binary trees, where instances are recursively partitioned and produce noticeable shorter paths for anomalies since: (i) in the regions occupied by anomalies, less anomalies result in a smaller number of partitions – shorter paths in a tree structure, and (ii) instances with distinguishable attribute-values are separated early in the partitioning process [34], [35], [36]. Given a feature vector x from a larger set of n-input feature vectors, say $X=\{x_{1},x_{2},\cdots ,x_{n}\}$, the anomaly score is defined as in [34], [36]:

(1) $$s\left(x,M\right)=2^{-\frac{E(h(x))}{c(M)}},$$

where M is the number of samples, c(M) the average path length of unsuccessful searches similar to the Binary Search Trees, h(x) is a harmonic number which is defined as ln(x) plus the Euler’s constant, and $E(h\left(x\right))$ is the average of h(x) from a collection of isolation forests. Samples with scores very close to 1 are marked as anomalies, whereas samples with scores smaller than 0.5 are inliers. The Local Outlier Factor (LOF) [34], [36] was also used as a density-based approach which measures the local density of a given data point with respect to its neighboring points, where the number of nearest neighbors determines the accuracy of the model. For a data point q∈x, the local reachability density of q, lrd(q), is defined as [36]:

(2) $$lrd\left(q\right)=\frac{\|N_{k}\left(q\right)\|}{\sum_{q\text{'}\in N_{k}\left(q\right)}r\left(q,q\text{'}\right)},$$

where $N_{k}\left(q\right)$ is the set of k-nearest neighbors for q, and $r(q,q^{\text{'}})$ is the reachability distance which is defined as the distance between x and its k-nearest neighbor.

##### 2.2.3.2 De-duplication

De-duplication is a critical stage of the data curator which involves the detection of highly correlated pairs of features, as well as, features with common sequences of characters. Towards this direction, the Spearman rank-order correlation coefficient was used to detect features with increased similarity in terms of distribution overlap. The Levenshtein distance score [34] was used to quantify the string similarity between each pair of features by calculating the edit distance between each pair of feature labels. The edit distance aims to calculate the number of different characters to transform one label into another by performing three types of operations, namely: (i) insertion, (ii) deletion, and (iii) substitution. Thus, the number of minimum operations determines the number of different characters among them and thus their lexical similarity.

##### 2.2.3.3 Final annotation and data quality approval

A data evaluation report was generated, where the available features within the raw cohort data were classified according to their quality status into three types, namely the: (i) “bad” features (having more than 50% missing values), (ii) “good” features (no missing values), and (iii) “fair” features (having less than 50% missing values). The “bad” features are excluded from the analysis. Features with detected outliers and/or unknown data types are marked to be excluded from the analysis. The cohort data curation workflow can be recursively applied until the cohort data quality metrics (completeness, conformity, and relevance) are fulfilled. The data evaluation report along with the curated cohort data were finally stored in the private cloud spaces which are linked with each individual cohort database.

### 2.3 Data sharing management module

The data sharing management module is responsible for: (i) handling the requests for cohort data access that are made by the data processors through the application of the data analytics services (this process is referred to as “handshaking”), and (ii) providing regular reports to the data providers regarding the usage of the cohort data. The “bring the analysis to the data” design is adopted according to which the data never leave from their private cloud spaces during when a data analytics workflow takes place. In the HarmonicSS platform, a federated data analytics workflow is executed only when the data provider approves the request that is sent by the data processor who invokes this workflow. Thus, when a data processor wishes to train a lymphoma classification model across multiple cohorts, the handshaking service informs, in real time, the data providers who own these cohort databases for this specific request. Thus, the data processor can only proceed with the analysis of those cohort databases whose data providers have approved this request.

### 2.4 Cohort data harmonization module

#### 2.4.1 Construction of the pSS reference ontology

The core of the cohort data harmonization process is based on the use of the pSS reference ontology [37]. The latter is a hierarchical data model which consists of classes, subclasses and object properties which capture the clinical domain knowledge of pSS. The reference ontology was constructed in cooperation with the clinical experts to reflect the minimal requirements of the pSS domain, i.e., a set of clinical, demographic, laboratory and therapeutic-related parameters which describe the inclusion criteria for pSS. The pSS reference ontology includes 5 main classes (i.e. demographics, therapies, biopsies, medical conditions, laboratory tests) with more than 150 terminologies and was expressed into a .RDF (Resource Description Framework)/.OWL (Web Ontology Language) format to enhance its sustainability and expandability for the easier integration with healthcare data management systems that support the HL7′s FHIR (Fast Healthcare Interoperability Resources) protocol [38].

#### 2.4.2 Semantic matching and data standardization

**Fig. 2:**
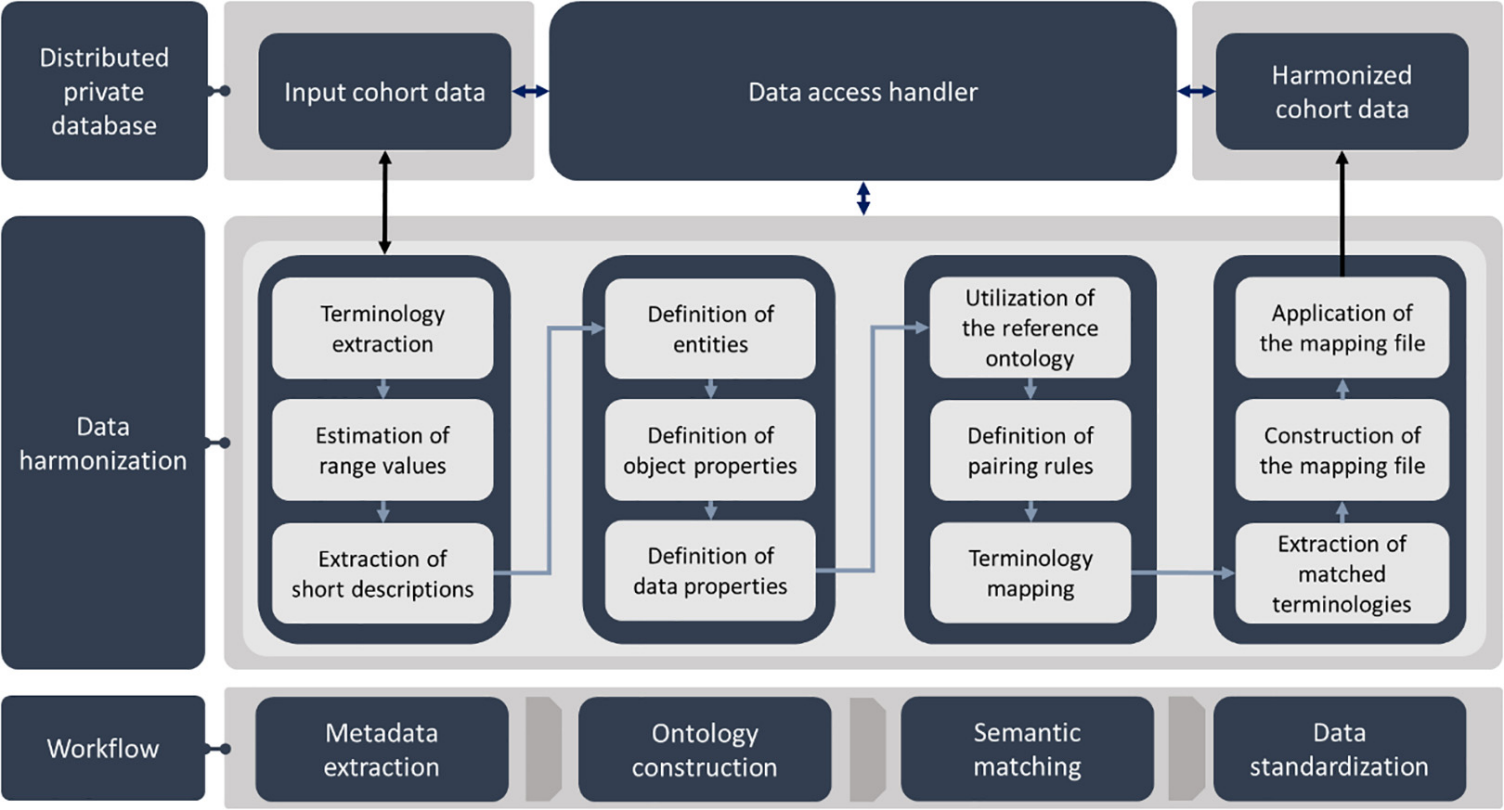
An illustration of the cohort data harmonization workflow.

Based on the metadata extracted from each cohort, an ontology was constructed to represent the structure of each cohort as an hierarchical data model (Fig. 2) through the definition of entities, data properties (data types) and object properties (hierarchical relationships), using Protégé [39]. The extracted metadata include information regarding the terminologies, the range values, and a short description of each concept. The pSS reference ontology from Section 2.4.1 was utilized as a gold-standard data model to align the structure and the range values of the individual ontologies. More specifically, the terminologies of the individual ontologies were semi-automatically matched with those from the pSS reference ontology through the definition of pairing rules according to clinical guidance, as well as, through the suggestion of relevant matches. Upon the precise definition of the pairing rules, the range value of each matched terminology was standardized through an additional computational procedure which involves the alignment of the heterogeneous value ranges in each individual ontology with the pre-defined range values in the pSS reference ontology which was expressed into the form of a mapping file (similar to a .log file) which was stored in the secure private cloud space of each cohort. The data access handler (Fig. 2) was used to monitor the data access as part of the data sharing management module (Section 2.3).

#### 2.4.3 Harmonized cohort data storage

In the final stage of the cohort data harmonization process, the mapping files of each individual cohort was used to align the structure of the cohort data yielding the harmonized cohort data (Fig. 2). The latter were stored in secure private cloud databases within the HarmonicSS cloud computing infrastructure. The private cloud databases are also referred to as federated databases. The latter were developed in SPARK-SQL (Structured Query Language) where secure communication protocols were established to enable easier data access and management under a virtual private network (VPN) connection.

### 2.5 Federated AI analytics module

#### 2.5.1 Federated AI framework

**Fig. 3:**
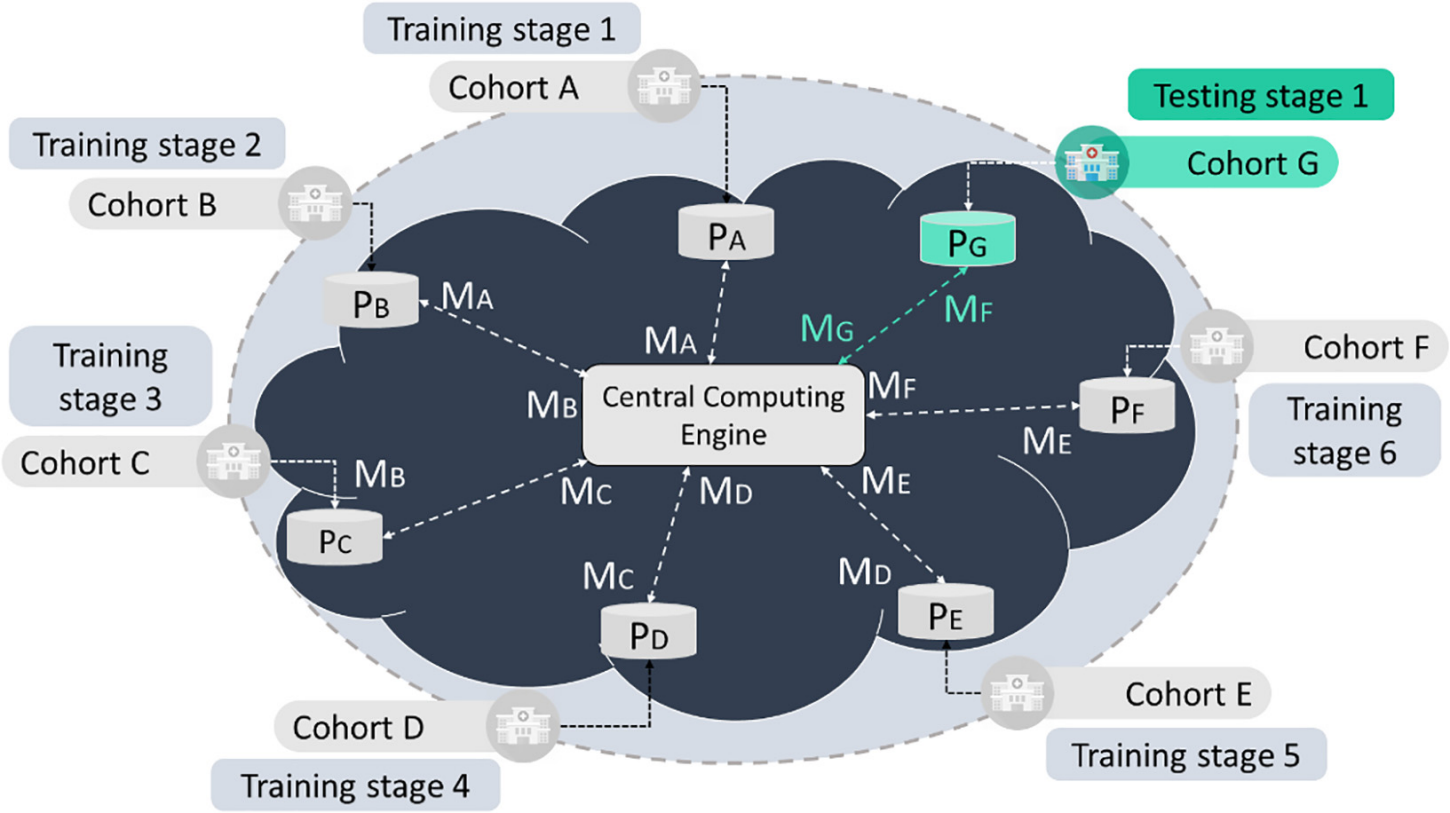
An illustration of the federated AI model training and testing workflow, where the testing cohort is depicted in green color. (For interpretation of the references to color in this figure legend, the reader is referred to the web version of this article.)

For a given a set of N-databases, say $\{D_{1},D_{2},\cdots ,D_{N}\},$ a machine learning algorithm trained on the dataset $d_{i}\in D_{i}$ is updated through the following function:

(4) $$F\left(d_{i}\right)=F\left(d_{i-1}\right)+\beta q\left(d_{i}\right),$$

where, $F\left(d_{i}\right)$ corresponds to the estimated ML model which has been trained on the dataset $d_{i}$, $F\left(d_{i-1}\right)$ corresponds to the estimated model which was trained on $d_{i-1}$, $q(d_{i})$ is the learner on $d_{j}$**,** and β is a scalar. A loss function can then be defined in the form $L\left(f\left(d_{i}\right),y_{i}\right)$ where $f\left(d_{i}\right)$ is the estimator and $y_{i}$ is the target score. Then, the stochastic gradient descent (SGD) approach [40] is used to minimize the loss function through the following sequential weight update process:

(5) $$w\left(d_{i}\right)=w\left(d_{i}-1\right)-\beta \left(\nabla_{w}L\left(f\left(d_{i}\right),y_{i}\right)+a\nabla_{w}r\left(w\right)\right),$$

where, $\nabla_{w}L\left(f\left(d_{i}\right),y_{i}\right)$ is the gradient of the loss function with respect to w, r(w) is a regularization function, $\nabla_{w}r\left(w\right)$ is the gradient of the regularization function, a is a hyperparameter, and β is a learning rate parameter. A pseudocode implementation of the incremental learning process across N-sites is presented in Algorithm 1. The algorithm uses as input a set of training cohorts, say train, a set of testing cohort(s), say test, and an initial supervised machine learning model (object), say M, which will be used for the design of federated AI models. To do so, a Central Computing Engine (CCE) is used to orchestrate the federated AI modeling training and testing procedure by incrementally transferring the weights of the AI model which is trained on the first training cohort to the rest of the training cohorts towards the extraction of the final AI model which is validated on a set of either one or more testing cohorts. For this purpose, the CCE was built on top of virtual machines (VMs) which were utilized in the GRNET cloud infrastructure to enable the secure access of the AI model’s weights on each cohort database. For demonstration purposes and according to Fig. 3, which depicts the federated AI model training and testing workflow, the set of training cohorts was defined as {A,B,C,D,E,F} and the testing cohort was set to {G}. The model M is first loaded into the Central Computing Engine (CCE) along with the set of training and testing cohorts. According to the workflow (Algorithm 1, Fig. 3), the model is trained on the dataset in location $P_{A}$ yielding the model $M_{A}$. The model’s weights are incrementally updated on the training cohorts which are stored in the private locations $P_{B},P_{C},P_{D},P_{E},P_{F}$ yielding the intermediate AI models $M_{B},M_{C},M_{D},M_{E},M_{F}$. During each iteration, the weights of each intermediate AI model are stored in the CCE. The model $M_{F}$ is then returned as the final federated AI model from the training stage which is used for the validation procedure in the cohorts which have been parsed as input in test.

**Table:** 

**Algorithm 1.** A pseudocode implementation of the federated AI modeling process.	
**Input parameters**	
train **=** a set of training cohorts which are stored in federated databases (and private cloud spaces)	
test **=** a set of testing cohort(s) which are stored in federated databases (and private cloud spaces)	
M **=** an initial supervised machine learning model	
1	**def federatedAImodeling (**$train=\left\{A,B,C,D,E,F\right\},test=\left\{G\right\},M$**):**
2	train the initial model M on the dataset in location $P_{A}$ and receive the model $M_{A}$
3	store the weights of $M_{A}$ in the Central Compute Engine (CCE)
4	for iintrain do:
5	retrieve weights and send them to location $P_{i}$
6	update the weights of $M_{i}$ on dataset in location $P_{i+1}$ through (5)
7	store the weights of the model $M_{i+1}$ in the CCE for the update process in the next location
8	retrieve the final federated model $M_{G}$ from the training stage
9	evaluate the performance of $M_{G}$ on the dataset in location $P_{G}$ (test)
10	return $M_{G}$;

An illustration of the federated AI workflow is depicted in Fig. 3.

#### 2.5.2 Federated AI algorithms

Federated stochastic gradient descent (FSGD) based classifiers

The incremental strategy which is adopted by the federated AI modeling process (Algorithm 1) offers a unique scalability which allows us to extend conventional supervised machine learning classifiers for federated learning tasks. More specifically, the loss function, $L\left(f\left(d_{i}\right),y_{i}\right)$, in (5) can be adjusted to build supervised machine learning classifiers for federated training and testing. To develop the federated logistic regression (FLR) classifier we can replace the regularization term in (3) with the logistic loss function:

(6) $$L\left(f\left(d_{i}\right),y_{i}\right)=ln\left(1+exp\left(-y_{i}f\left(d_{i}\right)\right)\right).$$

In a similar manner, we can develop the federated SVM (FSVM) algorithm using the hinge loss function:

(7) $$L\left(f\left(d_{i}\right),y_{i}\right)=max\left(0,1-y_{i}f\left(d_{i}\right)\right)$$

Finally, if we replace the loss function with the Perceptron loss:

(8) $$L\left(f\left(d_{i}\right),y_{i}\right)=max\left(0,-y_{i}f\left(d_{i}\right)\right)$$

we develop the federated Perceptron classifier and the federated Multi-layer Perceptron (FMLP).

Federated multinomial Naïve Bayes (FMNB)

In the case of discrete features, the multinomial Naïve Bayes (MNB) is preferred. Given an N-dimensional input vector, assume $d=(d_{1},d_{2},\cdots ,d_{N})$, where $d_{i}$ is the frequency of an event $e_{i}$, the class, say $c_{k}$, with the highest probability or the maximum a-posterior (MAP) class, can be solved as a linear function [41] using the logarithm expression:

(9) $$c_{\mathrm{MAP}}={\mathrm{argmax}}_{c_{k}}\left[\log\left(P\left(c_{k}\right)\right)+\sum_{i=1}^{N}\log\left(P\left(e_{i}|c_{k}\right)\right)\right].$$

where $P\left(e_{i}|c_{k}\right)$ is the conditional probability of the event $e_{i}$ given the class $c_{k}$, and k is the class index.

Federated gradient boosting trees (FGBTs) with and without dropouts

In the case of the gradient boosting trees (GBTs) schema, regression trees ensembles are used as weak learners to minimize the expected value of the loss function. In the case of the GBTs, we incrementally seek for the mapper F(x) at a stage m, $F_{m}\left(x\right)$ [42]:

(10) $$F_{m}\left(d_{i}\right)=F_{m-1}\left(d_{i}\right)+p_{m}h\left(d_{i};a_{m}\right),$$

where $p_{m}$ is the line search, and $h\left(x;a_{m}\right)$ is a regression tree learner with parameters $a_{m}$. A crucial problem in GBTs though is the fact that the trees added early in the ensemble tend to become more significant in the decision-making process than those added later. A solution to this issue is to use dropout rates [50], where the dropped trees and the newly added tree are scaled by a factor which ensures that the combination of the dropped trees and the new trees have the same effect on the outcome. To do so, the DART is trained on random subsets to prevent the definition of trivial trees. For a model, say Q, where Q(d) is the prediction for sample d, and $L\left(Q\left(d\right)\right)$ is the loss function DART creates the random subset [43]:

(11) $$\left\{\left(d,-\nabla_{t}L\left(Q\left(d\right)\right)\right)\right\},$$

where a new label with values $-\nabla_{d}L\left(Q\left(d\right)\right)$ is assigned for each sample d in the training dataset.

#### 2.5.3 Federated AI model explainability and interpretability

The SHapley Additive explanation analysis (SHAP) is a novel method from coalition game theory which can shed light into an AI model’s decision-making process [44]. To do so, SHAP utilizes explanation models that yield interpretable and explainable classification outcomes. Given a subset of input features, say $P\subset \{d_{1},d_{2},\cdots ,d_{Z}\}$, from a larger set of K-features $\left\{d_{1},d_{2},\cdots ,d_{K}\right\}$, where Z≤K, the SHAP value of a feature $d_{j}\in D$, say $S_{j}$, is defined as the overall contribution of this feature to the outcome, as in [44]:

(12) $$S_{j}=\sum \frac{\left|D\right|!\left(P-\left|D\right|-1\right)!}{P!}\left(f_{d}\left(D\cup \left\{d\right\}\right)-f_{d}\left(D\right)\right),$$

where, K is the set of all input features, $\left|D\right|$ is the number of features in D, and $f_{d}\left(D\right)$ is the expected value of the function conditioned on P. To deal with the computational burden introduced in Eq. (12), we adopt an estimation process [45] which reduces the complexity from $O(TL2^{Z})$ to $O(SLD^{2})$, where T is the number of trees, L is the total number of leaves, Z is the number of features, and D is the tree depth. The cover metric was also used to measure the number of observations which are related to a particular feature. For each feature, the relative number of observations is calculated as the number of splits that this feature participated across each ensemble and averaged across the training instances on each distributed database.

### 2.6 Visual analytics and user interfaces module

**Fig. 4:**
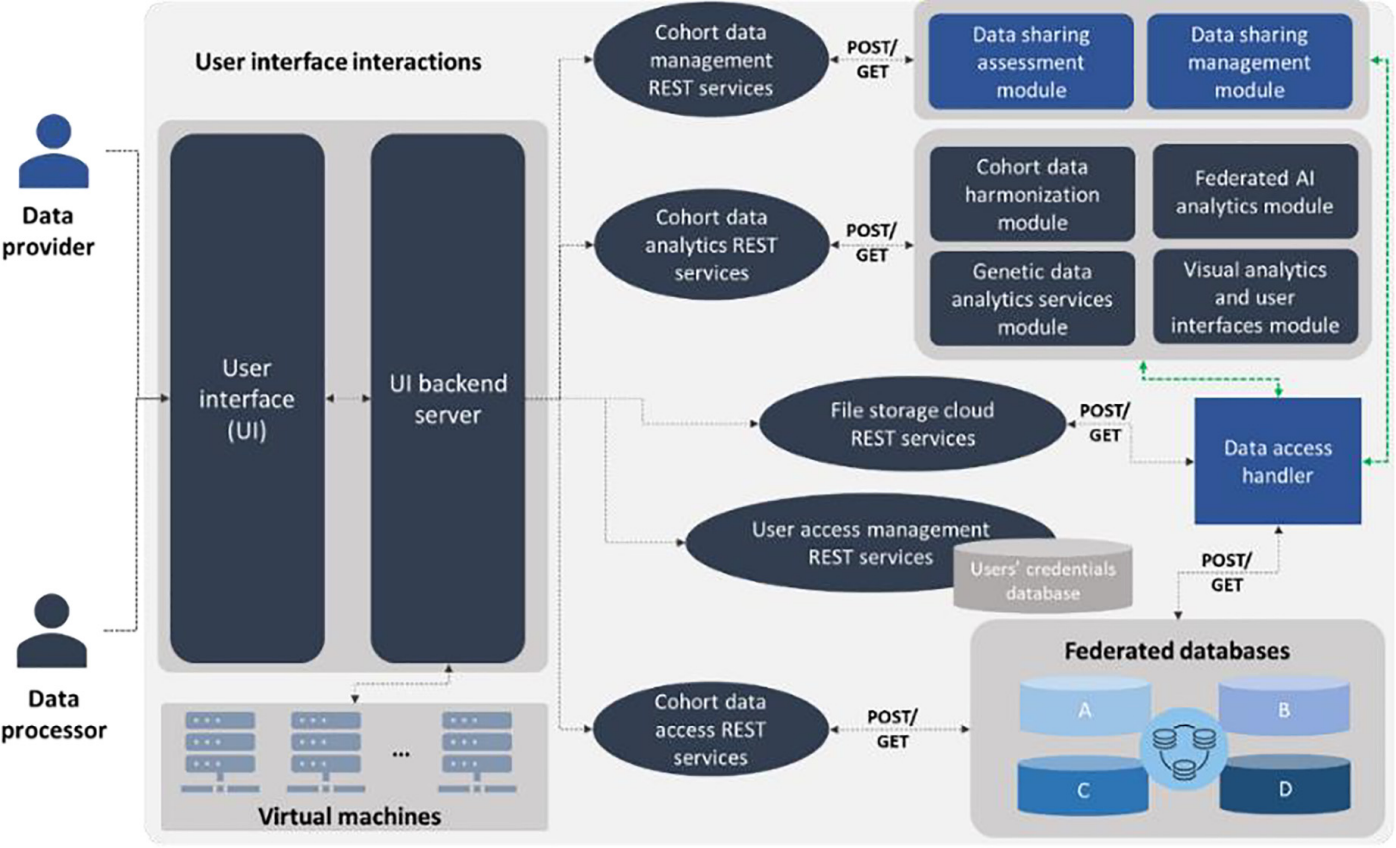
An illustration of the UI interactions within the HarmonicSS platform. Green arrows denote secure communication protocols. POST/GET commands refer to REST service requests. (For interpretation of the references to color in this figure legend, the reader is referred to the web version of this article.)

The visual analytics methods were implemented using the HealthVision web visualization platform [46] which consists of visualization and data analysis components that are linked to each other in reactive workflows. Each component accepts specific inputs, either from other components or from the user, and produces outputs that can be used by other components, or renders visual components (input controls, etc.) on the screen. The user interface (UI) serves as connecting link between the platform user and the backend services (Fig. 4). The contribution is shared between the actual UI client, and the UI backend server. The backend server is responsible for orchestrating: (i) access to the REST (Representational state transfer) services of other users, including user authentication, execution of data analytics services, data sharing management, etc. (ii) access to the cloud file storage, where the backend server handles file transfer to the cloud and manages directory structures for services that require file uploads via the user interface, (iii) access to the MySQL databases of prospective cohort data, where the structured data are stored in SQL (structured query language) tables with a common database schema setup. Semantic information, e.g. a patient’s laboratory test’s result, is also assembled by different queries across several tables.

## 3 Results

### 3.1 Cohort data origin

A summary of the overall demographic information from the 21 European databases on pSS is presented in Table 1. The total number of eligible patients who fulfilled the inclusion criteria was 7,156, where the gender information was recorded for 7,000 patients (6,512 females, 488 males with a female to male ratio 13.34%). The average age at SS diagnosis in the female group was 51.82 (± 13.96) years whereas in the male group the average age was 54.24 (± 13.77) years.

**Table 1:** Demographic information.

Demographics	Females	Males
Gender	6,512	488
Age at SS diagnosis (mean ± std)	51.82 (± 13.96) years	54.24 (± 13.77) years
Disease duration (mean)	7.08 years	5.59 years
Female to male ratio	13.34%	

The lymphoma types include the B-cell Mucosa-associated Lymphoid Tissue (MALT) Lymphoma, the Diffuse Large B-cell Lymphoma (DLBCL), the B-cell Nodal Marginal Zone Lymphoma (NMZL), the B-cell Splenic Marginal Zone Lymphoma (SMZL), and other mature B-cell neoplasms. These lymphoma types were merged into a single lymphoma type with 354 positive lymphoma patients and 6,802 non-lymphoma (or missing) patients (lymphoma to non-lymphoma ratio 5.2%). The lymphoma distribution per cohort is summarized in Table 2.

**Table 2:** Distribution of lymphoma and non-lymphoma patients per cohort.

Cohort acronym	Cohort full name	Number of lymphoma patients	Number of non-lymphoma (or missing) patients
IDIBAPS	Consorci Institut D’Investigacions Biomediques August Pi I Sunyer	0	300
UNIPG	Università degli Studi di Perugia	10	166
UPSUd PARIS	Université Paris-Sud (database 1)	24	483
UoB	University of Birmingham	3	156
UNIVAQ	Università degli Studi dell'Aquila	3	97
ULB	Université libre de Bruxelles	1	726
HUA	Harokopion University of Athens	8	151
UMCG	University Medical Center Groningen	20	166
UiB	University of Bergen	3	138
UOI	University of Ioannina	7	279
UU	Utrecht University	14	108
UNIRO	Universita' Degli Studi Di Roma La Sapienza	14	532
QMUL	Queen Mary University of London	1	47
UMCU	Universitair Medisch Centrum Utrecht	27	313
MHH	Medizinische Hochschule Hannover	5	178
UNIPI	Universita di Pisa	31	687
CUMB	Charité – Universitätsmedizin Berlin	0	71
UBO	Université de Bretagne Occidentale	4	77
UOA	National and Kapodistrian University of Athens	101	488
AOUD	Azienda Sanitaria Universitaria Integrata di Udine	16	281
UNEW	University of Newcastle	62	1358

### 3.2 High quality and harmonized cohort databases

Data curation was applied on each individual cohort database to automatically remove outliers, data inconsistencies and duplicated fields. The LOF algorithm was combined with the Isolation Forests to track down and remove outliers with 90% accuracy and the Spearman correlation coefficient was combined with the Jaro distance score to detect duplicated features. Data imputation was applied only to features with less than 30% missing values as in [47] upon approval from the clinical experts. Upon the completion of the cohort data curation process, ontologies were constructed for each curated cohort database based on the extracted metadata. Semantic mapping rules were defined between the individual ontologies and the pSS reference ontology. As shown on Supplementary Table 1, the cohort data harmonization process resulted in 48 common concepts (or terminological concepts) which constitute the pSS minimal criteria (minimal common data elements) across the 21 federated cohort databases.

### 3.3 Federated AI models for lymphoma classification and biomarker extraction

According to Table 2, the lymphoma over non-lymphoma ratio was 5.2% which implies a significant population imbalance. To deal with this, random downsampling with replacement [15] was applied on each individual training cohort database among the lymphoma (target group) and the non-lymphoma (control group) patients. The process was repeated ten times to avoid biases during the downsampling process. On each iteration, the downsampled control group was matched with the target group according to the age, gender, and disease duration using a ratio 1:1 to yield equally balanced populations. The Wilcoxon Mann-Whitney rank-sum test was used to evaluate whether the distributions of the age and disease duration did not significantly deviate between the target group and the downsampled control group whereas the chi-square test was used for gender matching. The classification performance of the federated AI models was assessed based on the accuracy, sensitivity, specificity, and area under the ROC curve (AUC).

Four large scale federated lymphoma classification scenarios were conducted; three scenarios including a common set of training harmonized cohort databases and three different testing databases, as well as, one scenario with a different set of training databases and a single testing database. The training set in federated scenarios 1–3 is {UOA, UNIPI, UNEW, UNIPG, PARIS, UoB, UNIVAQ, HUA, UOI, UU, UNIRO, UMCU, MHH, UBO} and the testing set is {AOUD (scenario 1), UNIPG (scenario 2), HUA (scenario 3)} whereas the training set in federated scenario 4 is {AOUD, UOA, UNIPI, UNIPG, UNEW, PARIS, UoB, UNIVAQ, UOI, UU, UNIRO, UMCU, MHH, UBO, UMCU} and the testing set is HUA. According to Table 3, the federated tree ensembles achieved better performance against the FSGD-based methods, such as, the FMNB and the FMLP, since the latter focus on the direct update of the weights of a linear loss function, without controlling for overfitting effects, their performance tends to be lower than in the case of the federated tree ensembles which utilize boosting to avoid overfitting.

**Table 3:** A summary of the performance evaluation results across the four federated scenarios.

Federated learning schema	Performance evaluation metrics			
	Accuracy	Sensitivity	Specificity	AUC
**Federated scenario 1**				
**FGBT**	0.84	0.81	0.85	0.89
**FDART, rd = 0.1**	0.86	0.75	0.87	0.87
**FDART, rd = 0.2**	0.84	0.62	0.85	0.86
**FDART, rd = 0.3**	0.83	0.81	0.84	0.89
FDART, rd = 0.4*	0.85	0.81	0.85	0.89
**FDART, rd = 0.5**	0.83	0.87	0.83	0.88
**FMNB**	0.51	0.94	0.49	0.71
**FMLP**	0.64	0.75	0.63	0.69
**Federated scenario 2**				
**FGBT**	0.71	0.70	0.71	0.73
**FDART, rd = 0.1**	0.69	0.70	0.69	0.76
FDART, rd = 0.2*	0.74	0.80	0.73	0.79
**FDART, rd = 0.3**	0.71	0.70	0.71	0.71
**FDART, rd = 0.4**	0.71	0.70	0.71	0.75
**FDART, rd = 0.5**	0.71	0.70	0.71	0.76
**FMNB**	0.63	0.70	0.63	0.66
**FMLP**	0.68	0.70	0.68	0.69
**Federated scenario 3**				
**FGBT**	0.75	0.99	0.74	0.89
FDART, rd = 0.1*	0.78	0.99	0.76	0.90
FDART, rd = 0.2*	0.78	0.99	0.76	0.91
**FDART, rd = 0.3**	0.76	0.99	0.74	0.90
**FDART, rd = 0.4**	0.71	0.87	0.69	0.86
**FDART, rd = 0.5**	0.74	0.75	0.74	0.86
**FMNB**	0.71	0.87	0.70	0.79
**FMLP**	0.85	0.62	0.87	0.74
**Federated scenario 4**				
**FGBT**	0.81	0.75	0.81	0.91
**FDART, rd = 0.1**	0.78	0.87	0.78	0.92
**FDART, rd = 0.2**	0.80	0.75	0.80	0.91
FDART, rd = 0.3*	0.80	0.87	0.79	0.91
**FDART, rd = 0.4**	0.80	0.87	0.80	0.90
**FDART, rd = 0.5**	0.78	0.75	0.78	0.91
**FMNB**	0.62	0.87	0.61	0.74
**FMLP**	0.85	0.62	0.86	0.74

* With light blue color: The federated schema with the best performance, rd: dropout rate.

**Fig. 5:**
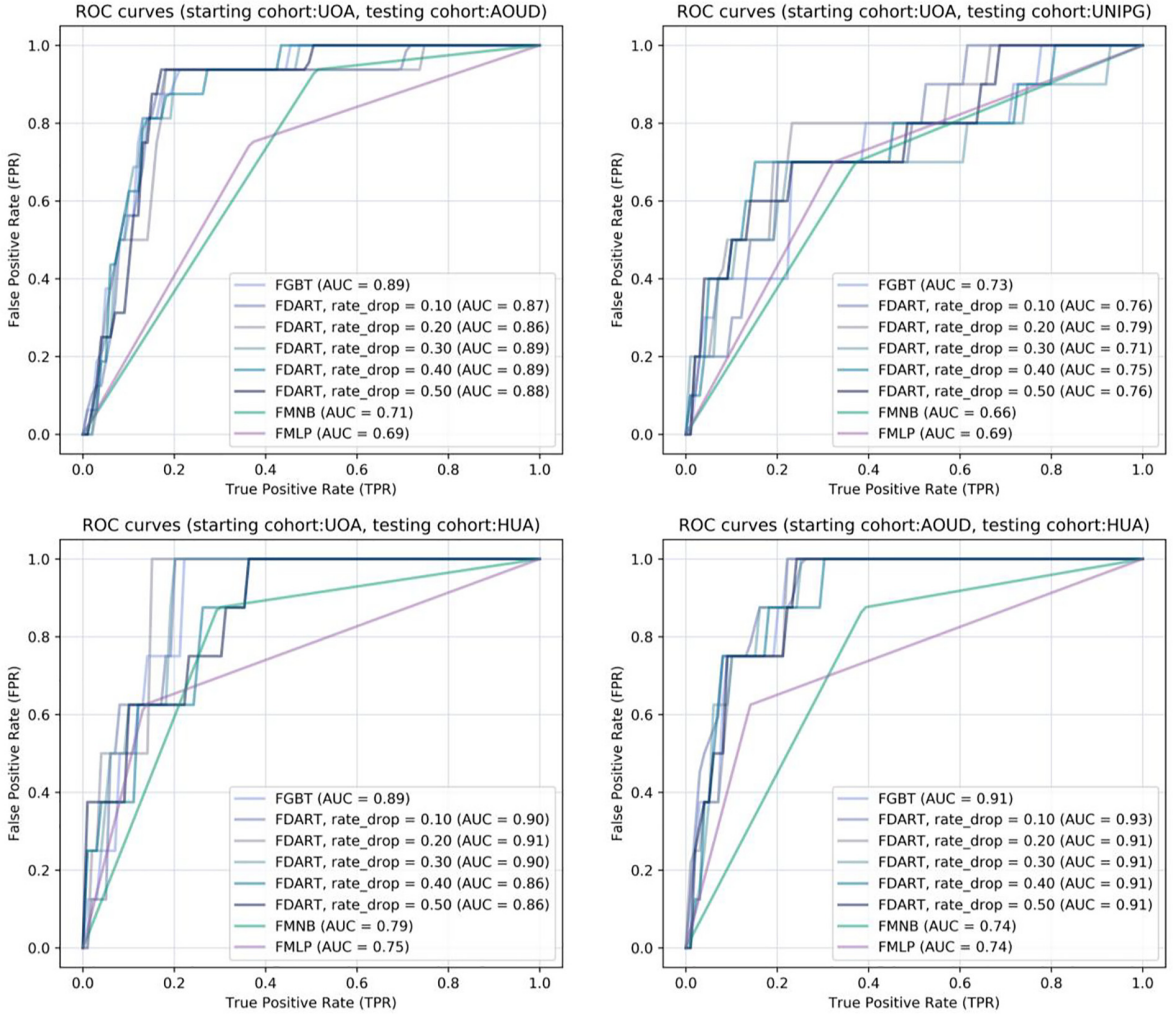
Receiver Operating Characteristic (ROC) curves for each federated algorithm across the two federated scenarios. From top to bottom: on the left for federated scenario 1 with testing cohorts AOUD, UNIPG, HUA and for federated scenario 2 testing cohort HUA.

According to Fig. 5, the ROC curves confirm the favorable performance of the FDART along with the FGBTs, in all cases, where the FDART with dropout rate 0.4 achieved the best performance in federated scenario 1 (accuracy 0.85, sensitivity 0.81, specificity 0.85). Regarding federated scenario 2, the FDART with dropout rate 0.2 achieved the best performance (accuracy 0.74, sensitivity 0.8, specificity 0.73). In federated scenario 4, the FDART with dropout rates 0.1 and 0.2 achieved the best performance (accuracy 0.78, sensitivity1, specificity 0.76) like the FGBT (accuracy 0.75, sensitivity 1, specificity 0.74). In the final scenario, the FDART with dropout rate 0.3 achieved the best performance (accuracy 0.8, sensitivity 0.87, specificity 0.79) yielding better sensitivity than the FGBTs, where the average execution time was 30 s for data access and training/testing on each harmonized cohort database.

### 3.4 Biomarkers for lymphoma classification and federated AI model explainability

**Fig. 6:**
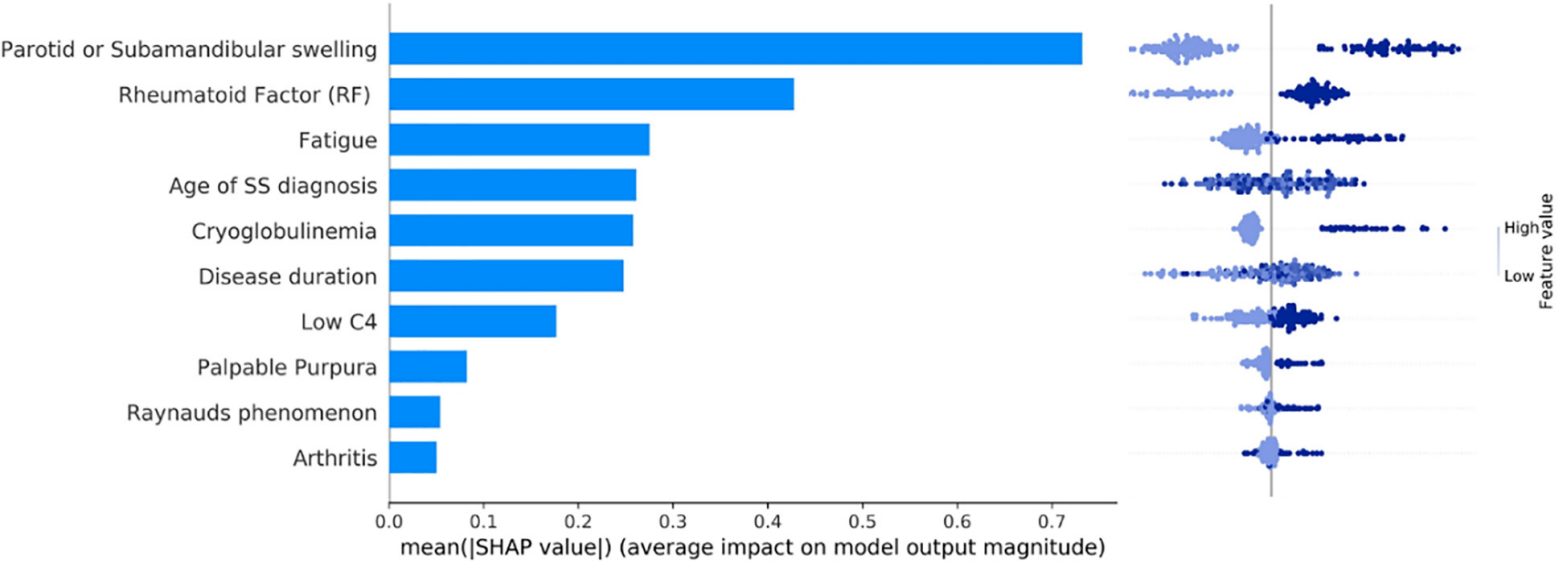
An illustration of the SHAP plot in federated scenario 1 for the FGBT.

**Fig. 7:**
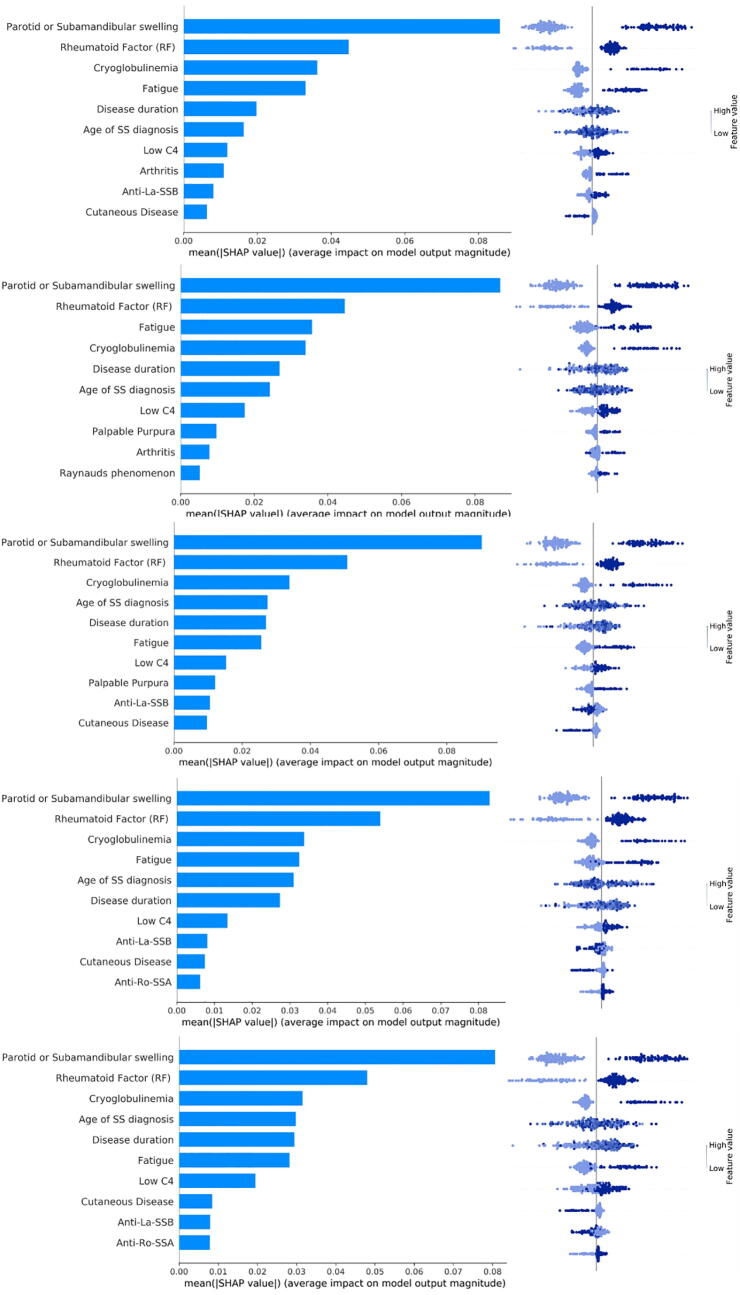
An illustration of the SHAP plot in federated scenario 1 for the FDART schemas.

The results of the Shapley additive explanation analysis are depicted in Fig. 6 for the FGBT classifier and in Fig. 7 for the FDART classifier with dropout rates 0.1–0.5, where the features are ranked based on their positive or negative impact on lymphoma development. Each panel in Fig. 6 reflects the mean Shapley value (i.e., the average of the marginal contributions across all permutations) for a feature, in descending order, as well as, whether the impact of a feature has a positive (left) or a negative (right) value for lymphoma development. In Fig. 6, Fig. 7, the color in the distribution plots denotes whether the importance of the Shapley value is either low or high and the vertical line corresponds to the base score of the AI model centered around zero.

According to Fig. 6 and Fig. 7, the feature “Parotid or Submandibular swelling” has the highest impact in lymphoma classification, where its absence has a negative predictive value and thus decreases the risk for lymphoma development whereas its presence has a positive predictive value on lymphoma development (i.e., the positive samples shift the ground truth to the right). Features “Rheumatoid factor”, “Fatigue”, “Age of SS diagnosis”, “Cryoglobulinemia”, and “Disease duration” come next with favorable impact on lymphoma classification. Features “Low C4”, “Palpable purpura”, “Raynaud's phenomenon”, “Arthritis” also appear to be significant in the decision-making process. The importance of these features is also confirmed by the average coverage of each federated tree ensemble algorithm during the lymphoma decision-making process (Supplementary Fig. 1).

The Shapley explanation analysis results for the federated learning scenarios 2, 3, and 4 are depicted in Supplementary Fig. 3, Fig. 4, Fig. 5, Fig. 6, Fig. 7, for the FDART and FGBT, respectively. According to Fig. 6 and Fig. 7, the features “Parotid or Submandibular swelling”, “Rheumatoid factor”, “Cryoglobulinemia”, “Age at SS diagnosis”, “Fatigue”, and “Low C4” appear to be prominent for lymphoma classification. In all cases, patients with parotid or submandibular swelling, rheumatoid factor, cryoglobulinemia, fatigue and Low C4 tend to have higher impact for lymphoma development since the positive samples shift the ground truth to the right, thus yielding a positive contribution to lymphoma development. The same effect occurs in the case where the pSS spatients exhibit palpable purpura, Raynaud’s phenomenon, and arthritis, as well (Fig. 6).

## 4 Discussion

The HarmonicSS cloud computing services delineated the clinical picture and unmet needs of pSS through: (i) the utilization of a unique data governance framework that enables the extensive evaluation of the DPA and DPIA documents by the Data Controller’s Committee (DCC) and the secure upload of the GDPR compliant cohort data in federated databases, (ii) the application of cohort data curation and harmonization workflows on 21 regional, national and international European cohorts on pSS yielding 7,156 high quality patient records, and (iii) the utilization of high-performance federated AI workflows towards the development of explainable and trustworthy federated AI models for lymphoma classification and biomarker extraction. The data sharing assessment module can also support cross border data sharing since it ensures: (i) the secure upload of the legally and ethically compliant data on the federated databases of the platform using secure data encryption protocols, and (ii) the secure access of the data analytics services on the data through the handshaking protocol towards the development of trustworthy AI models.

The existing platforms and tools that have been developed for data curation, harmonization and federated or distributed data analysis are presented in Supplementary Table 2 and compared against the core services of the HarmonicSS platform. The existing studies for data curation focus on the development of software tools, such as, the ExeTera software [16], where the absence of quantitative methods for data curation along with the lack of re-usable quality reports hampers the generalizability of the software. In addition, the DPUK [17] adopts a qualitative approach based on quality criteria that are manually defined for each individual data source. In HarmonicSS, quantitative data curation tools have been developed to enhance the quality of the data and provide re-usable reports to the clinicians. Regarding data harmonization, the DataSHaPER [18] utilizes ontologies based on the definition of a DataSchema which is not widely used as a semantic data model. The BiobankConnect software [19] focuses on lexical matching which can lead to information loss when the terminologies are conceptually similar. The SORTA tool [20] focuses on the alignment of heterogeneous ontologies through manual semantic interlinking methods. Contrary to these, the HarmonicSS platform offers a cohort data harmonization service which uses lexical and semantic matching to identify terminologies with common lexical and conceptual basis, where the pSS reference model is expressed into a .RDF/.OWL format. As far as federated/distributed learning is concerned, the euroCAT platform [21], [22] requires the installation of local servers on each hospital’s premises, where the distributed learning algorithms include Bayesian networks and Support Vector Machines which were trained across 3 centers to predict dyspnea yielding modest prediction performance. In the PHT platform [23], a distributed logistic regression model was trained across 8 sites to predict post-treatment with adequate performance. In other studies, lymphoma classification models were trained across 4 pSS cohorts for federated lymphoma classification [15] and single cohorts were used to develop lymphoma classification models with reduced statistical power [11], [12], [13]. The HarmonicSS platform removes the need for the installation of local servers or any type of software on premises through the development of a federated data management system that supports a large family of federated AI algorithms with small execution time complexity yielding robust and explainable AI models for lymphoma classification. In addition, the HarmonicSS platform is cloud agnostic and thus can be adapted to any cloud infrastructure.

The automated data curation workflow enhanced the quality of the raw cohort data at a great extent and the cohort data harmonization module enabled the application of semantic interlinking mechanisms on each curated cohort database yielding harmonized cohort databases with 7,156 patients. The federated AI services provided trustworthy and explainable AI models for lymphoma classification and biomarker(s) detection, where the federated AI modeling process is orchestrated by the central computing engine (CCE). The access of the CCE to each individual harmonized cohort database is monitored by the data sharing management module to enhance the trustworthiness of the AI models. So far, the platform supports six algorithms for federated learning, including the federated logistic regression, the federated SVM, the federated MLP, the federated Multinomial Naïve Bayes (FMNB), the federated gradient boosting trees (FGBT), and the FGBTs with and without dropout elimination (FDART). The FGBT and the FDART achieved the best performance for lymphoma classification due to the robustness of the boosting stages which reduce the classification error on each stage. The FDART outperformed the rest of the algorithms yielding lymphoma classification models with average AUC 0.87 across the scenarios. The dropout rates introduced by the FDART yielded slightly better performance than the FGBT which confirms that the dropout elimination can enhance the decision-making process. The execution time of the federated AI workflows was 30 s (in average) per database which confirms the small execution time complexity.

The biomarkers for lymphoma development include parotid or submandibular swelling, cryoglobulinemia, rheumatoid factor, and low C4 levels, among others, which have been validated in previous studies [48], [49], [50] highlighting the significance of parotid or submandibular gland swelling, low C4, rheumatoid factor and cryoglobulinemia for lymphoma development. In [48], [49] salivary gland swelling and cryoglobulinemia appear to be significantly higher in pSS patients evolving into lymphoma compared to pSS controls. In fact, cryoglobulinemia can affect many extraglandular organs, such as, the kidney, the skin, and the peripheral nerves, leading to permanent damage. The impact of age of SS diagnosis was also highlighted as a prominent factor in [9], [10], where the time interval from pSS diagnosis to lymphoma has been stated as a biomarker for lymphoma prediction. Furthermore, patients with the presence of parotid or submandibular swelling, rheumatoid factor (RF), cryoglobulinemia, and low C4 tend to have higher impact for lymphoma development. This can be confirmed by the distribution of the samples in Fig. 6 and Fig. 7 which shift the ground truth to the right direction and thus have a positive predictive value for lymphoma development.

The federated AI workflows which are offered by the platform are built on top of a federated repository on autoimmune disease data along with the data curation and harmonization services for enhancing the quality of the cohort data. These services are executed under a PaaS (Platform as a Service) cloud computing model, which enhances the sustainability of the platform based on three trajectories: (i) the maintenance of the PaaS operations from a legal and ethical point of view, (ii) the collaboration among multidisciplinary and international partners able to attract funds and investments from sponsors, and (iii) the implementation of a business model. The fact that the HarmonicSS platform is compliant with HL7 standards enhances its applicability to other clinical domains. The federated data analytics services of the platform can be applied only on harmonized databases and thus emphasis shall be given towards the definition of an ontology for the domain of interest. Apart from the core modules though, the HarmonicSS platform provides services for health policies impact assessment, association rule mining, and query-based knowledge discovery, as well as, tools for salivary gland ultrasonography image segmentation and patient selection for multinational clinical trials, and training material for both clinicians and patient organizations. The platform can also offer, upon request to the DCC, the option for the extraction of the anonymized harmonized data to provide the clinicians with the opportunity to apply statistical analysis and related approaches. The federated AI model can be used for the accurate risk prediction of lymphoma and thus contribute to the early lymphoma diagnosis in patients who have been diagnosed with pSS avoiding additional costs for biopsies. In addition, the AI model provides explainable scores which can be used by the clinician to assess the contribution of critical risk factors for lymphoma development and thus support the clinical decision-making process. The impaired 10-year survival of SS patients with MALT lymphomas and the association of lymphoma stage with the overall prognosis, point out the necessity for early lymphoma diagnosis and thus the development for lymphoma prediction models [51], [52]. As a future work, we plan to further enhance the performance of the federated AI model by including genetic data (e.g., FMS-like tyrosine kinase 3 ligand).

## Declaration of Competing Interest

The authors declare that they have no known competing financial interests or personal relationships that could have appeared to influence the work reported in this paper.
