# Supplementary material for: Addressing the clinical unmet needs in primary Sjögren’s Syndrome through the sharing, harmonization and federated analysis of 21 European cohorts
Source: Comput Struct Biotechnol J. 2022 Jan 7;20:471–84. doi: 10.1016/j.csbj.2022.01.002 (PMC8760551; doi:10.1016/j.csbj.2022.01.002)
Supplement: Supplementary data 1 [file mmc1.pdf]

Supplementary Material

Supplementary Tables

Supplementary Table 1: Set of features which represent the minimal criteria of the pSS domain knowledge.

**Feature**	**Presence/ Abnormal**	**Absence/ Normal**	**Mean**	**Median**
Gender	6879 (females)	513 (males)	-	1
Age at SS diagnosis	-	-	51,95	53
Disease duration	-	-	7,23	6
Dry Mouth (aka Xerostomia)	6101	699	-	1
Dry Eyes	6046	748	-	1
Parotid or Submandibular swelling	1897	3186	-	0
Parotid Gland swelling	1616	2227	-	0
Submandibular salivary gland swelling	139	2648	-	0
Raynaud's Phenomenon	1577	4365	-	0
Fatigue	2840	2416	-	1
Arthritis	993	5006	-	0
Renal Disease	162	6021	-	0
Tubulointerstitial Nephritis	66	4663	-	0
Glomerulopathy	37	4293	-	0
Membranoproliferative Glomerulonephritis (MPGN)	15	4309	-	0
Membranous Glomerulonephritis (MGN)	3	4163	-	0
Mesangioproliferative Glomerulonephritis (MPGN)	9	4157	-	0
Other Glomerulonephritis	4	4501	-	0
Pulmonary Disease	415	5421	-	0
Small Airway Disease	157	5012	-	0
Lymphocytic Interstitial Pneumonia (LIP)	47	4414	-	0
Nonspecific Interstitial Pneumonia (NSIP)	34	4034	-	0
Usual Interstitial Pneumonia (UIP)	31	4041	-	0
Cryptogenic Organizing Pneumonia (COP)	0	4077	-	0

Liver Disease	131	5300	-	0
Autoimmune Hepatitis (AIH)	40	4682	-	0
Primary Biliary Cholangitis (PBC)	81	5535	-	0
Sclerosing cholangitis	11	4639	-	0
Nervous System Disease	560	5577	-	0
Peripheral Nervous System Disease	267	4712	-	0
Central Nervous System Disease (CNS)	125	4487	-	0
PalpablePurpura	396	5257	-	0
CutaneousDisease	458	4158	-	0
Muscular System Disease	357	4633	-	0
IdiopathicInflammatoryMyopathy (IIM)	10	3842	-	0
Inclusion Body Myositis (IBM) documented with Biopsy	150	4429	-	0
B-cell Mucosa-associated Lymphoid Tissue (MALT) Lymphoma	245	5324	-	0
Diffuse Large B-cell Lymphoma (DLBCL)	45	5326	-	0
B-cell Nodal Marginal Zone Lymphoma (NMZL)	24	5346	-	0
B-cell Splenic Marginal Zone Lymphoma (SMZL)	6	4789	-	0
Other mature B-cell neoplasms	21	5305		0
Anti-La-SSB [presence]	2670	3499	--	0
Anti-Ro-SSA [presence]	4565	1703	-	1
Rheumatoid Factor (RF) [Units-volume]	2282	2362	-	0
Antinuclear Antibodies (ANA) [presence]	4354	1096	-	1
C4 levels (Serum complement) [Mass-volume]	2485	1725	-	1
Cryoglobulins [presence]	266	4409	-	0
Lymphoma*	354	5653	-	0

* The records of patients with missing lymphoma status were ignored from the analysis.

Supplementary Table 2: Comparison of the HarmonicSS platform with the other state-of-the art platforms and tools for data curation, data harmonization and federated/distributed learning.

Study	Category	Scope/Rationale	Outcome	Comparison
Murray et al., 2020 [15]	Data curation	To present an open-source data curation software designed to address scalability across the COVID Symptom Study dataset.	The ExeTera software provides functionality that enables a data curation pipeline incorporating data curation methods for COVID. The pipeline includes preliminary data cleaning and filtering using semantic information, and generation of meta-analytics for daily assessment.	• Lack of quantitative methods for data quality control.<br>• Lack of re-usable quality reports.<br>• Focuses on a particular data schema for semantic matching of existing information.
Bauermeister et al., 2020 [17]	Data curation	Present a platform for data curation, data discovery, access brokerage, data analysis and knowledge preservation	The raw data are curated to a common data model (C-Surv). The C-Surv ontology is designed to simplify the analytic challenge of working across multiple datasets and multiple modalities by providing standard structure, variable naming, and value labelling conventions.	• The quality assessment process is exclusively based on quality criteria that are manually defined for each individual data source.<br>• Lack of quantitative methods for data curation.
Fortier et al., 2011 (DataSHAPER) [18]	Data harmonization	Uses a DataSchema as a reference model to harmonize heterogeneous data schemas according to the	A 36% compatibility for creating a harmonized database across 53 of the world's largest longitudinal population-based	• Small percentage of matched terminologies.<br>• The input ontology is exclusively based on the definition

		user-defined DataSchema through the development of pairing rules.	epidemiological studies.	of a DataSchema which is not a widely used semantic data model.
Pang et al., 2015 (Biobank Connect) [19]	Data harmonization	Uses lexical and semantic matching to align heterogeneous biobanks according to a desired set of pre-defined elements.	An average precision 0.745 towards the harmonization of data across six biobanks (7,461 terms) with 32 desired elements.	• Lexical matching can lead to information loss in the case where the terminologies share a common conceptual basis.<br>• Focuses only on biobanks.
Pang et al., 2015 (SORTA) [20]	Data harmonization	Uses lexical matching to align phenotype data from heterogeneous biobanks according to international coding systems.	Matched 5210 entries in the LifeLines biobank (97% recall) and 315 entries in the DUMR (58% recall).	• Lexical matching can lead to information loss in the case where the terminologies share a common conceptual basis.<br>• Focuses only on biobanks.
Deist et al., 2017 (euroCAT) [21]	Federated / distributed learning	Distributed learning methodology for privacy-preserving multi-centric rapid learning.	Support vector machines (SVM) & the Alternating Direction Method of Multipliers (ADMM) for privacy-preserving multi-centric rapid learning across three clinical centers from five countries.	• The discriminative performance in the cross-validation is modest with a validation AUC of 0.66.<br>• Training AUCs are stable across folds (0.60–0.64) while inter-fold validation AUCs vary considerably (0.57–0.77).<br>• Requires the installation of a local server in each hospital.<br>• Small number of participating hospitals.
Jochems et al., 2017 (euroCAT) [22]			A Bayesian network model is adapted for distributed learning to predict dyspnea as a common side effect	• The AUC of the model is 0.61 (95%CI, 0.51–0.70) on a 5-fold cross-validation

			after radiotherapy treatment of lung cancer across three clinical centers from five countries.	and ranges from 0.59 to 0.71 on external validation sets.<br>• Requires the installation of a local server in each hospital.<br>• Small number of participating hospitals.<br>• Conventional machine learning methods for prediction.
Beyan et al., 2020 (Personal Health Train) [23]	Federated learning	To provide a privacy-by-design infrastructure connecting FAIR (Findable, Accessible, Interoperable, Reusable) data sources and allows distributed data analysis and machine learning. Patient data never leaves a healthcare institute.	A distributed logistic regression model predicting post-treatment two-year survival was trained on 14,810 patients treated between 1978 and 2011 and validated on 8,393 patients treated between 2012 and 2015.	• Software was installed locally to enable deployment of distributed machine learning algorithms via a central server.<br>• Average AUC 0.71 across 8 sites.<br>• Conventional machine learning methods for prediction.
HarmonicSS platform	Data curation, data harmonization, federated learning	To develop a platform which will provide a single point access to data curation and data harmonization services along with federated AI services to address the clinical unmet needs in pSS.	A total number of 7,551 high quality and harmonized patient data from 21 European cohorts. Federated AI models for lymphoma classification and lymphomagenesis with more than 0.9 AUC. Five prominent biomarkers for lymphoma development.	• Offers fully automated data curation workflows.<br>• Data harmonization is based on the definition of widely used semantic data models which are expressed in known formats (e.g., .RDF, .OWL format).

				• Avoids the installation of local servers or software in each hospital through the adoption of federated data management.<br>• Federated AI modeling for lymphoma classification across 21 federated databases in less than 1 minute.<br>• Offers interpretable and explainable AI models.<br>• Supports a large family of federated AI algorithms.<br>• Small execution time complexity.

Supplementary Figures

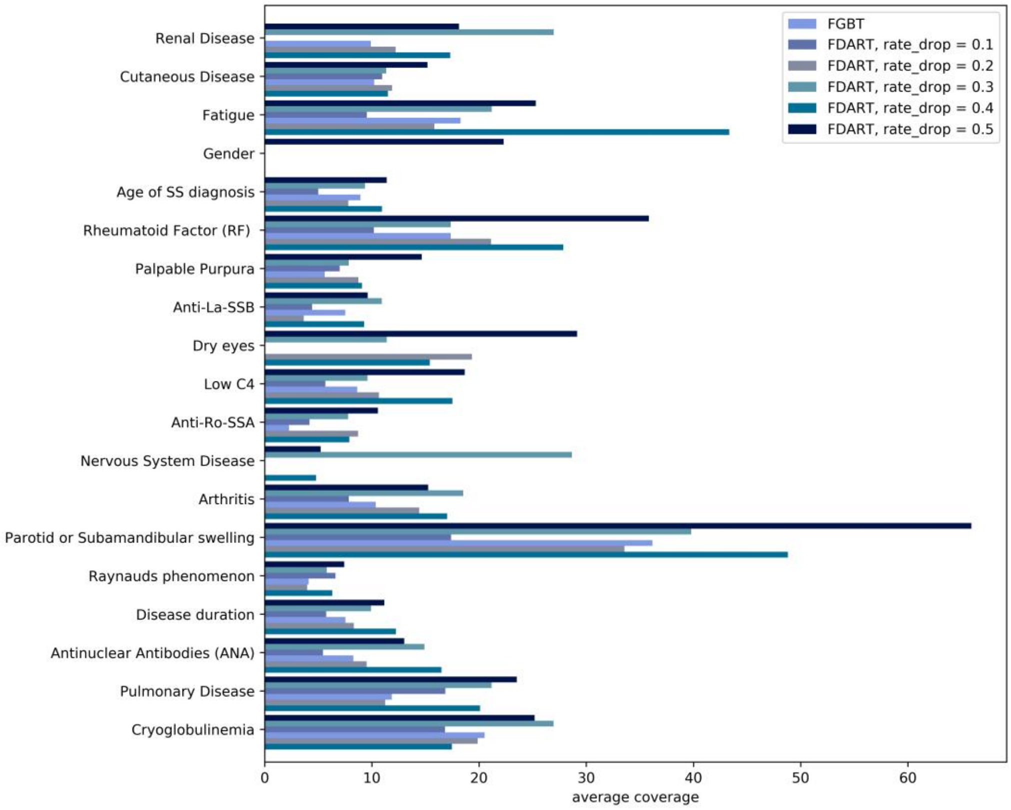

Supplementary Figure 1: The average coverage for each federated tree ensemble algorithm in federated scenario 1 which quantifies the average number of observations that passed through this feature (node) during the node splitting process.

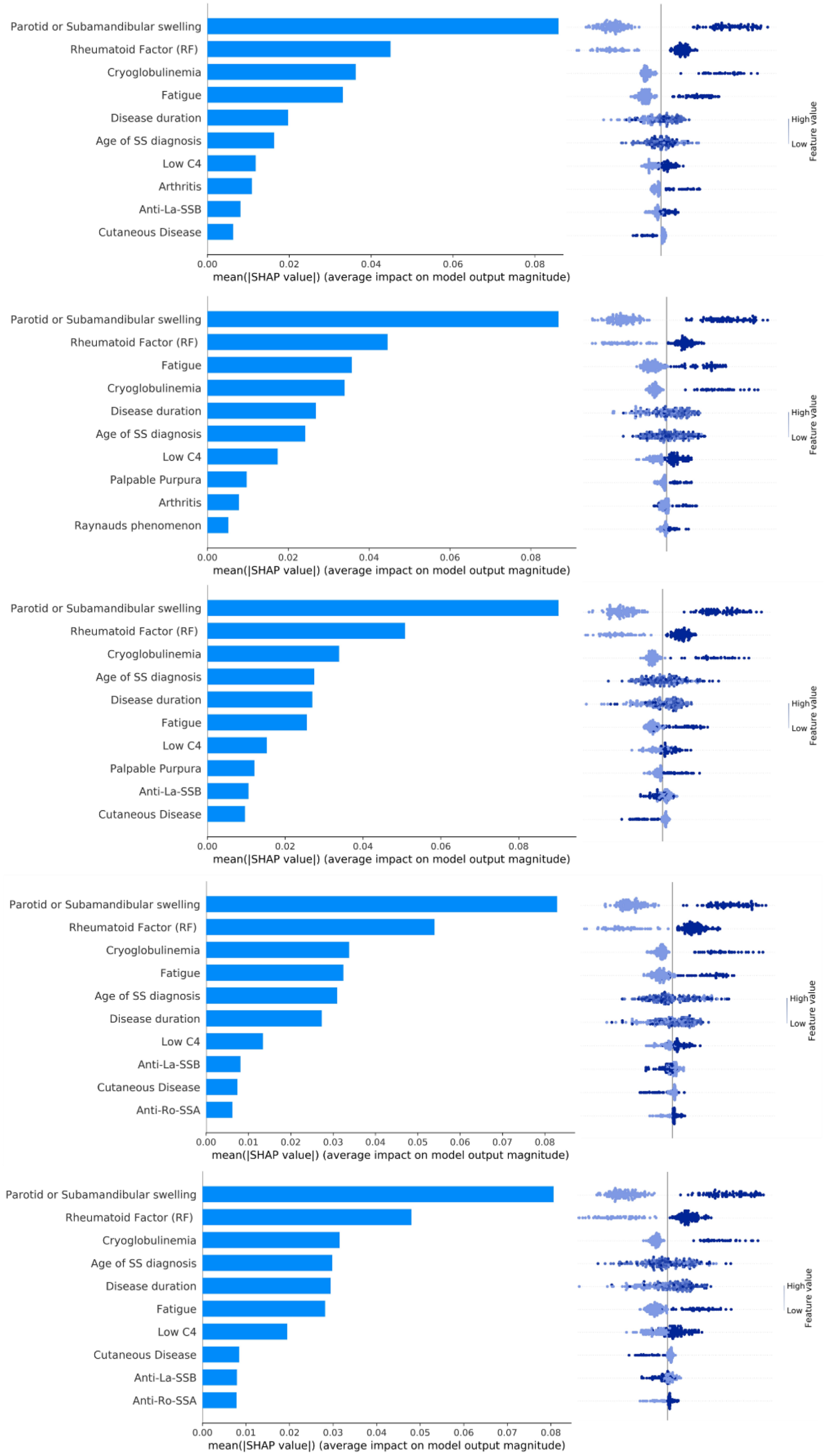

Supplementary Figure 2: An illustration of the SHAP plot in federated scenario 2 for the FDART schemas.

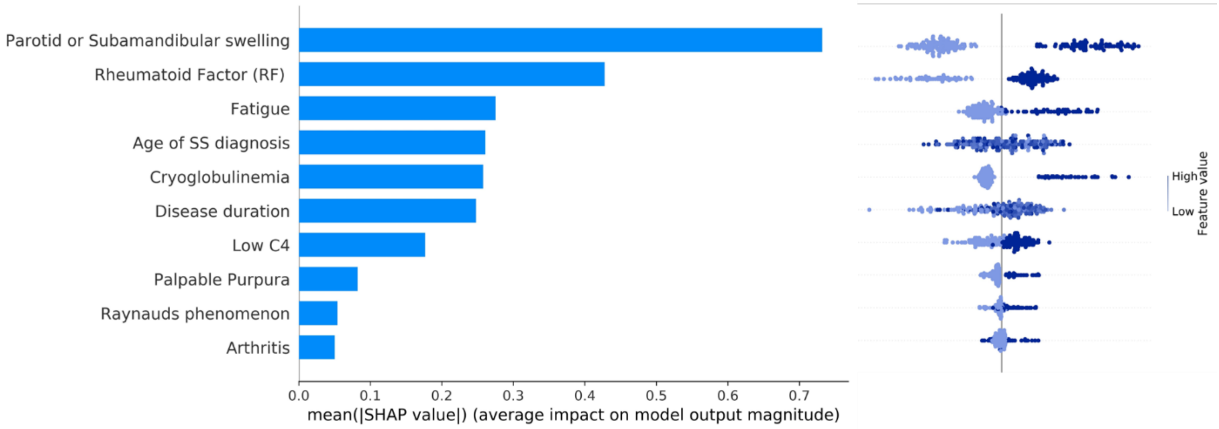

Supplementary Figure 3: An illustration of the SHAP plot in federated scenario 2 for the FDART schemas.

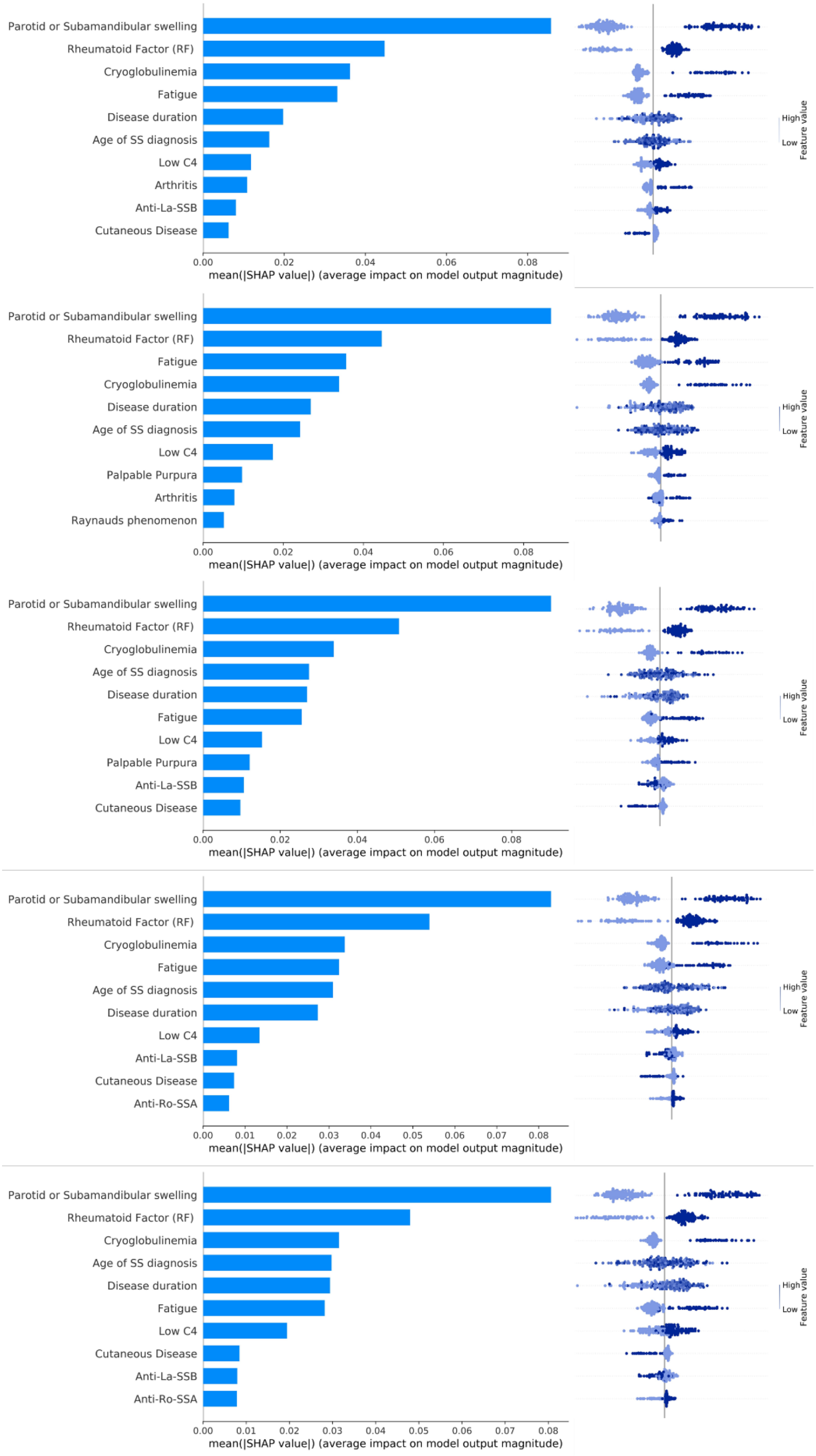

Supplementary Figure 4: An illustration of the SHAP plot in federated scenario 3 for the FDART schemas.

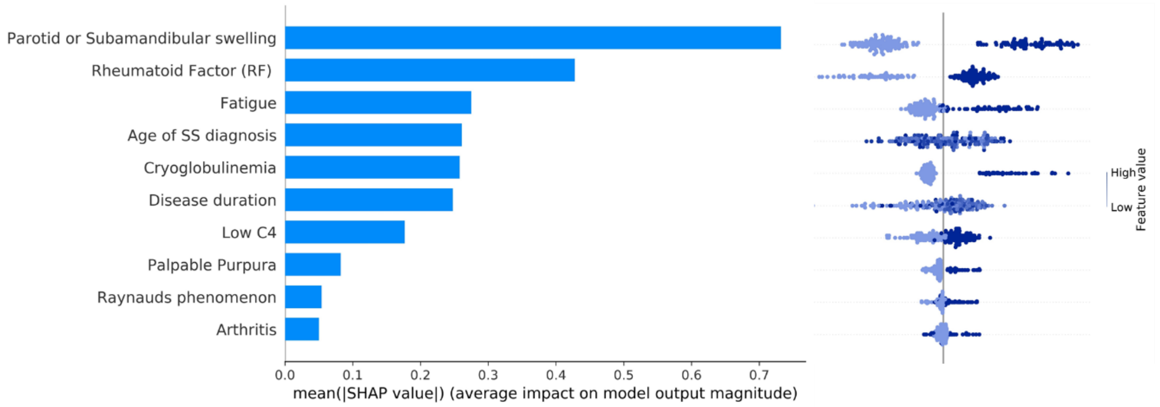

Supplementary Figure 5: An illustration of the SHAP plot in federated scenario 3 for the FDART schemas.

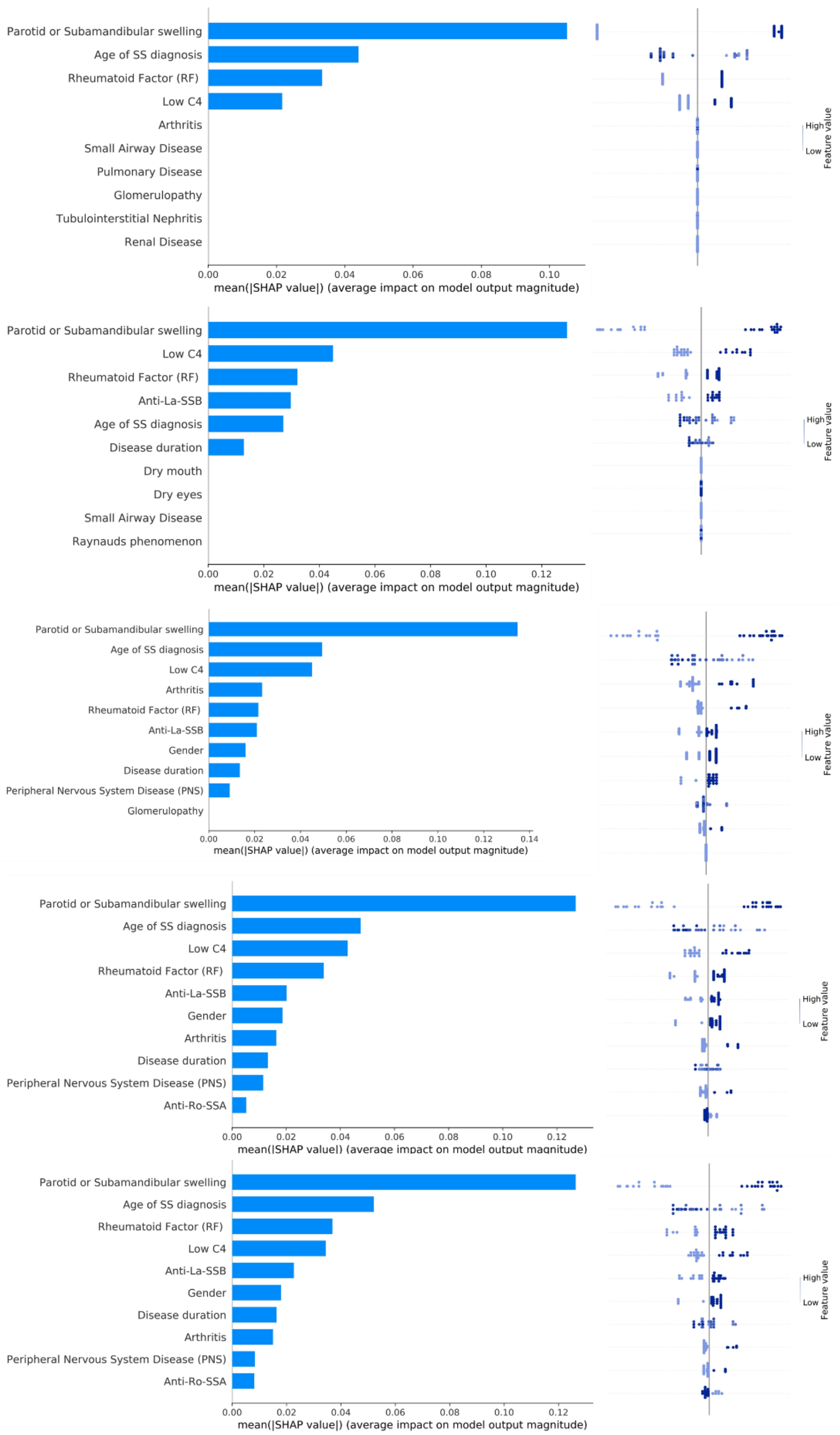

Supplementary Figure 6: An illustration of the SHAP plot in federated scenario 4 for the FDART schemas.

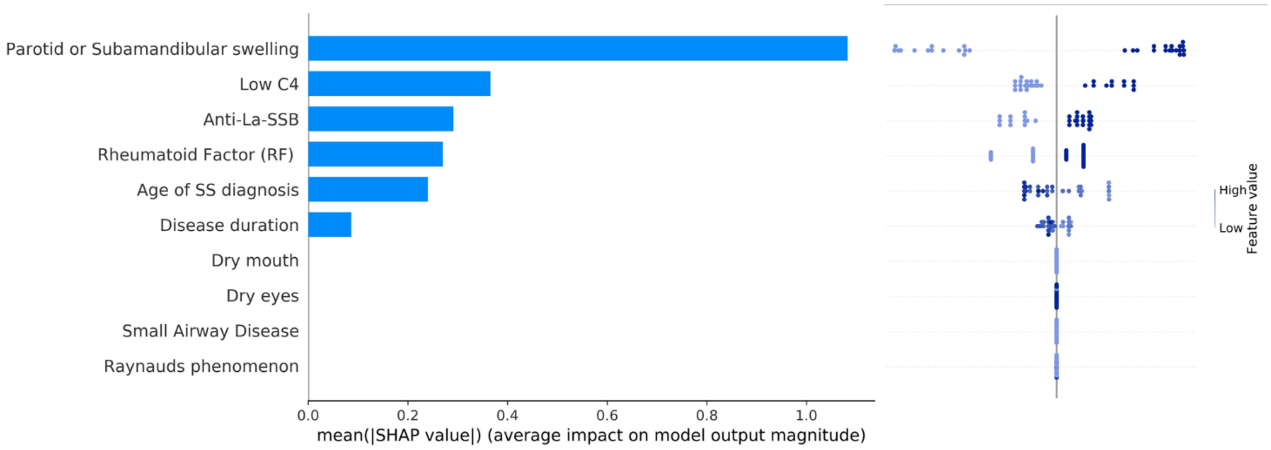

Supplementary Figure 7: An illustration of the SHAP plot in federated scenario 4 for the FDART schemas.
